# Comparative Dynamic Transcriptome Reveals the Delayed Secondary-Cell-Wall Thickening Results in Altered Lint Percentage and Fiber Elongation in a Chromosomal Segment Substitution Line of Cotton (*Gossypium hirsutum* L.)

**DOI:** 10.3389/fpls.2021.756434

**Published:** 2021-10-25

**Authors:** Yang Gao, Yu Chen, Zhangqiang Song, Jingxia Zhang, Wanyu Lv, Han Zhao, Xuehan Huo, Ling Zheng, Furong Wang, Jun Zhang, Tianzhen Zhang

**Affiliations:** ^1^State Key Laboratory of Crop Genetics and Germplasm Enhancement, Nanjing Agricultural University, Nanjing, China; ^2^Key Laboratory of Cotton Breeding and Cultivation in Huang-Huai-Hai Plain, Ministry of Agriculture and Rural Affairs, Cotton Research Center, Shandong Academy of Agricultural Sciences, Jinan, China; ^3^College of Life Sciences, Shandong Normal University, Jinan, China; ^4^Zhejiang Provincial Key Laboratory of Crop Genetic Resources, Institute of Crop Science, College of Agriculture and Biotechnology, Plant Precision Breeding Academy, Zhejiang University, Hangzhou, China

**Keywords:** lint percentage, single chromosomal segment substitution line, transcriptome analysis, secondary-cell-wall thickening, candidate genes

## Abstract

Lint percentage (LP) is an important yield component in cotton that is usually affected by initial fiber number and cell wall thickness. To explore how fiber cell wall development affects LP, phenotypic identification and dynamic transcriptome analysis were conducted using a single segment substitution line of chromosome 15 (SL15) that harbors a major quantitative trait locus (QTL) for LP. Compared to its recurrent parent LMY22, SL15 did not differ in initial fiber number, but the fiber cell wall thickness and single-fiber weight decreased significantly, altering LP. The comparative transcriptome profiles revealed that the secondary cell wall (SCW) development phase of SL15 was relatively delayed. Meanwhile, the expression of genes related to cell expansion decreased more slightly in SL15 with fiber development, resulting in relatively higher expression at SL15_25D than at LMY22_25D. SCW development-related genes, such as *GhNAC*s and *GhMYB*s, in the putative NAC-MYB-CESA network differentially expressed at SL15_25D, along with the lower expression of *CESA6, CSLC12*, and *CSLA2*. The substituted chromosomal interval was further investigated, and found 6 of 146 candidate genes were differentially expressed in all four cell development periods including 10, 15, 20 and 25 DPA. Genetic variation and co-expression analysis showed that *GH_D01G0052, GH_D01G0099, GH_D01G0100*, and *GH_D01G0140* may be important candidate genes associated with *qLP-C15-1*. Our results provide novel insights into cell wall development and its relationship with LP, which is beneficial for lint yield and fiber quality improvement.

## Introduction

Cotton (*Gossypium* spp.) is a principal source of natural fiber. To fulfill the fiber needs of the ever-increasing global population, it is essential to improve cotton yield (Tilman et al., 2011). Cotton lint yield is constituted of three important components: boll number, seed cotton weight per boll, and lint percentage (LP, determined as lint weight [LW, g]/seed cotton weight [g] × 100). Of these components, LP is notable for being more stable across different environments (Muhammad et al., 2006; Su et al., 2016), and is an important component for cotton yield (Wang et al., 2014). Several studies on improving LP have taken a quantitative genetics approach, identifying associated quantitative trait locus (QTLs) in different populations (Yu et al., 2013; Wang et al., 2014, 2016). Recent rapid developments in sequencing technology have enabled several *Gossypium* genomes to be sequenced and reported in succession: *G. raimondii* (Wang et al., 2012), *G. arboretum* (Li et al., 2014), *G. hirsutum* (Li F. et al., 2015; Zhang et al., 2015; Hu et al., 2019), and *G. barbadense* (Liu et al., 2015; Wang M. et al., 2019). Consequently, additional genome-wide association studies (GWASs) have been carried out to identify stable QTLs or critical genes associated with agronomic traits of interest (Fang et al., 2017b; Sun et al., 2018; Song et al., 2019; Su et al., 2019), thus identifying several candidate genes having functional roles closely related to LP (Su et al., 2016, 2019; Sun et al., 2018).

Cotton fiber cells serve as an effective single-cell system for studying cell wall development (Haigler et al., 2012). Fiber cell development is roughly divided into four overlapping growth phases: initiation, elongation, secondary cell wall (SCW) cellulose deposition, and maturity. LP is mainly determined by two main factors, the number of initiation fibers on the ovule epidermis and SCW thickness that correspond to the phases of fiber initiation and SCW biosynthesis, respectively. Fiber initiation takes place between −2 and 2 days post-anthesis (DPA) (Zhang et al., 2017a), and phytohormones play an important role in this process. For instance, indole-3-acetic acid is a major auxin that accumulates in fiber cells and positively regulates fiber initiation (Zhang et al., 2017a,b), while cytokinins negatively regulate fiber initiation by exerting an antagonistic effect on auxin accumulation on the ovule epidermis (Zeng et al., 2019). Transcription factors are also critical during the initiation of fiber development. For example, MYB-MIXTA-like transcription factors have been identified to orchestrate epidermal cell differentiation (Haigler et al., 2009; Wu et al., 2018), and several R2R3-MYB transcription factors are responsible for cotton fiber initiation; of those, *GhMYB25-like* has been identified as playing a key role in the early stage of fiber cell differentiation (Walford et al., 2011), while *GhMYB109*, homologous to *AtMYBGL1*, is specifically responsible for fiber initiation and elongation (Suo et al., 2003).

In contrast to common plant cell walls in which elongation and secondary wall thickening are independent events, the development of cotton fiber SCW is a unique process comprising two overlapping phases: cell elongation and wall thickening (Schubert et al., 1973). The deposition of cellulose determines fiber SCW thickness, with mature cotton fibers containing over 90% crystalline cellulose. Endogenous gibberellins have been found to play a critical role in cotton fiber SCW biosynthesis, as manipulation of their levels by transgenic methods led to significant increases in 1,000-fiber weight, cell wall thickness, and cellulose content (Bai et al., 2014). Sucrose is a major carbon source for cellulose biosynthesis, and the novel sucrose synthase encoded by *GhSusA1* has been identified as being tightly associated with cotton fiber yield (Jiang et al., 2012). Furthermore, several genes involved in SCW deposition have been identified and validated. When the R2R3-MYB transcription factor *GhMYBL1* is overexpressed, SCW-related genes are upregulated, resulting in increased cellulose and lignin biosynthesis (Sun et al., 2015). Another transcription factor, *GhXLIM6*, was found to promote cellulose biosynthesis by negatively regulating *GhKNL1* expression, thus subsequently affecting the expression of cellulose synthase A (CESA) genes (Li et al., 2018). Overall, it is well-established that cotton fiber cellulose deposition and SCW thickness are closely related to LP. However, few studies have investigated the mechanisms underlying the direct relationship between fiber cell wall thickness and LP.

In our previous study, a QTL cluster associated with fiber strength, fiber elongation and lint percentage was identified on chromosome 15 (Wang et al., 2020). Subsequently, a single chromosomal segment substitution line (CSSL) containing this QTL cluster (hereinafter referred to as SL15) was developed through multigenerational backcrossing with LMY22, a cultivar with a high lint percentage that is of low quality. In this study, the fiber–related trait phenotype of SL15 was characterized, and the transcriptome profile of SL15 together with its recurrent parent LMY22 was also analyzed. Transcriptional analysis revealed dynamic differences between SL15 and LMY22 in expression patterns and regulatory networks relevant to fiber cell wall development; in particular, this study identified co-expressed genes that were predominantly or specifically expressed in some stages of fiber development. The results would be beneficial for better understanding the biological/molecular mechanisms underlying fiber SCW thickening, as well as its most relevant LP.

## Materials and Methods

### Plant Materials

Two upland cotton lines with significant differences in LP, SL15 (low-LP), and LMY22 (high-LP), were used in this study. SL15 was developed from a set of single chromosomal segment substitution lines using LMY22 (recurrent parent) and LY343 (donor parent), meaning these lines are nearly isogenic.

All of the materials were grown under standard field conditions at the Linqing Experimental Station, Shandong Cotton Research Center (LES/SCRC) and Hainan Island in the winter during 2016–2019. LP was assessed by conventional cotton breeding methods. Fiber quality parameters were evaluated by the Supervision, Inspection, and Test Center of Cotton Quality, Ministry of Agriculture of China (Anyang, Henan Province) using a high-volume precision instrument. In addition, other yield data, namely boll weight, seed index, and seed number per boll, were precisely weighed and counted. The mean fiber length was detected by the Supervision, Inspection, and Test Center of Cotton Quality, Ministry of Agriculture of China (Anyang, Henan Province, China) using an AFIS single fiber test. Individual flower buds from each line were tagged at 0 days post-anthesis (DPA). Cotton fiber samples were collected at 10, 15, 20, and 25 DPA and frozen in liquid nitrogen for RNA-seq analysis, with at least 5–10 bolls harvested at each time point for two biological replicates; samples were labeled per line as LMY22_10D to LMY22_25D and SL15_10D to SL15_25D.

### Microscopic Observation of Fiber Initiation and Cell Wall Thickness

To observe the initiation of fiber cells, bolls were collected at 0 DPA from a similar position in LMY22 and SL15. The ovules were carefully taken from the same position of each boll and fixed in 2.5% (v/v) glutaraldehyde at 4°C. After a series of dehydration treatments and drying (Hu et al., 2016), the ovules were sprayed with gold powder by a Cressington 108auto. Fiber initiation was observed and photographed by a Hitachi TM3030 (Japan).

For the determination of cell wall thickness, cotton fibers collected at the last stage of SCW thickening (30 and 35 DPA) were fixed for 12 h at 25°C in 2.5% glutaraldehyde. After a series of washes, dehydration, and infiltration, the samples were embedded in resin for 48 h at 60°C; then, the middle parts of the fibers were cut into 6 μm sections. The slices were observed under a microscope (Leica RM2235), and cell wall thickness was surveyed using a ZEISS Axio Scope. A1. ZEISS software ZEN (blue edition) was employed to measure the thickness of cell walls, and approximately 100 fibers were measured for each sample.

### Determination of Single Fiber Weight and Fuzz Content

Fibers on seeds were combed straight and striped manually. The cotton fiber was cut (1 cm) in the middle part, and ~2 mg (W1) was used to precisely count the number of mature fibers. Those fibers were scattered over black cloth as much as possible, for photography, and the counting function of Adobe Photoshop CS6 was used to precisely count the number of fibers of 1 cm length (N1). The number of fibers per unit weight was calculated as (N2) = (N1×10)/(the mean fiber length of LMY22 and SL15, respectively). The weight of single fibers (W2) was calculated from the following equation: W2 = W1/N2. Fuzz content (%) = (weight of seeds–weight of delinted seeds)/weight of seeds ×100 as described (Zhang et al., 2011). Each set of data was calculated with 50 sample repeats.

### RNA Extraction, Library Construction, and RNA-Seq Analysis

Total RNA was extracted from each tissue sample using an RNAprep Pure Plant Kit (Huayueyang, Beijing, China) and quantified using a NanoDrop 2000 spectrophotometer (Thermo Scrientific, Waltham, Massachusetts, USA). RNA degradation and contamination were evaluated using 1% agarose gel electrophoresis. Meanwhile, RNA integrity was confirmed by an Agilent 2100 Bioanlyzer (Agilent Technologies, Santa Clara, California, USA). Finally, the 16 cDNA libraries were constructed, and sequencing was performed (Novogene Company, Tianjin, China) using the Illumina system (Illumina, San Diego, USA), generating 125/150 bp paired-end raw reads.

Clean data (clean reads) were obtained from raw data by removing adapters, reads containing poly-N stretches, and low-quality reads. At the same time, Q20, Q30, and GC content were determined for the clean data. Hisat2 v2.0.5 was used to build an index of the *G. hirsutum* reference genome (Hu et al., 2019), downloaded from Zhejiang University, as well as for mapping the clean reads to the genome. The mapped output was processed *via* feature Counts v1.5.0-p3 (Liao et al., 2014) to obtain FPKM for all transcript sequences in each sample. Correlations between biological replicates were determined via calculating Spearman's correlation coefficient (SCC).

### Differentially Expressed Gene Analysis and Comparison of Gene Expression Patterns

Differentially expressed genes (DEGs) were identified based on counts using the *DESeq2* R package. Genes were considered to be differentially expressed if they had FPKM > 0.5, false discovery rate <0.01, and|log2 (fold change)|>1. To reveal the biological processes in which these differentially expressed genes might be involved, Gene Ontology (GO) and Kyoto Encyclopedia of Genes and Genomes (KEGG) enrichment analyses were carried out using the *clusterProfiler* R package and corresponding databases. Terms or pathways with a corrected *P* < 0.05 were considered significantly enriched in DEGs. The expression patterns of DEGs were investigated using the *cluster* R package to assess whether the different stages of fiber development might be regulated by different DEG sets.

### Verification of DEGs by Quantitative Real-Time PCR

Total RNA was extracted from samples using the Plant RNA Kit, and cDNA was generated by reverse transcription with HiScript III RT SuperMix for qPCR with gDNA wiper (Vazyme, Nanjing, China) following the manufacturer's instructions. Quantitative reverse transcription PCR (qRT-PCR) was performed on a Light Cycler 480II (Roche, Germany) using SYBG Premix Ex Taq II (TaKaRa Bio, Kusatsu, Japan). *GhHistone3* (AF024716) (Xu et al., 2004) and *GhUBI1* (EU604080) (Zhang et al., 2018) were used as internal reference genes. The gene-specific and internal control primers are listed in Supplementary Table 1. Three biological and technical replications were performed in all qRT-PCR assays. The relative expression of differentially expressed genes was calculated by the 2^−ΔΔCt^ method.

## Results

### Phenotypic Characteristics of SL15

The substitution line SL15 was developed from a cross between LMY22 (high lint percentage) and LY343 (high quality), followed by continuous backcrossing with LMY22. To identify the genetic background of SL15, 307 simple-sequence repeat (SSR) markers that were evenly distributed across 26 chromosomes were selected based on a high-density linkage map of LMY22 × LY343 (Wang et al., 2013; Song et al., 2020). The results showed that 98.97% of the genetic composition of SL15 originated from LMY22, while the remaining 1.03% was from Chr. 15 of LY343, suggesting that the genetic background of SL15 was almost the same as LMY22 except for the segment of Chr. 15 (Supplementary Figure 1).

**Figure 1:**
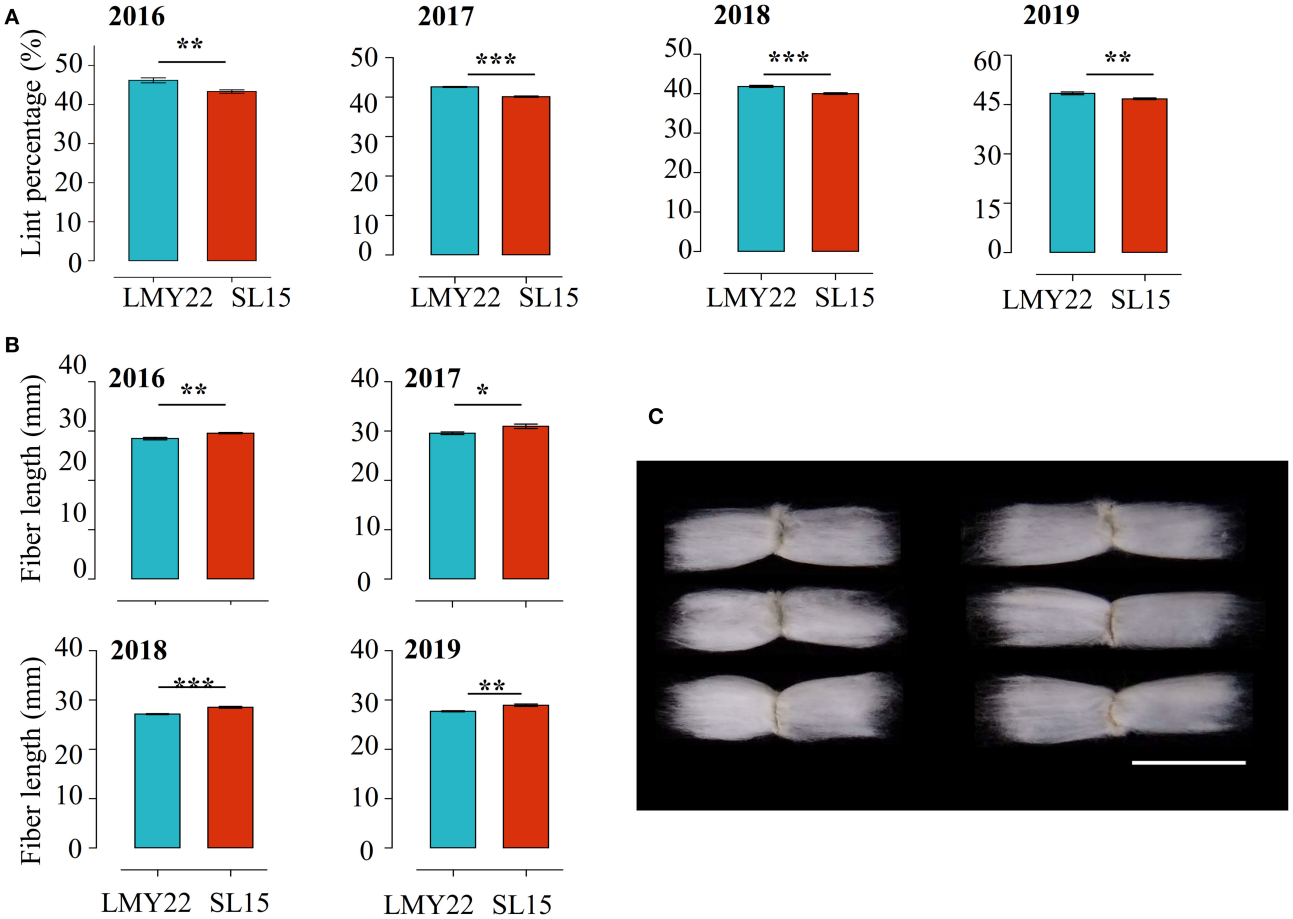
Multi-year field data comparisons. **(A)** Lint percentage and **(B)** fiber length in LMY22 and SL15 during a four-year field trial from 2016 to 2019 (mean ± s.d., *n* = 5, *P < 0.05, **P < 0.01, ***P < 0.001). **(C)** Representative fiber images from LMY22 (left) and SL15 (right). Scale bar, 3 cm.

The key agronomic traits of SL15 were investigated and no differences in plant architecture, leaf shape, and boll shape were observed between SL15 and LMY22. The yield-related traits, including boll weight, seed index, and seed number per boll, also had no significant variation (Supplementary Figures 2, 3). However, a 4-year field trial showed that the LP of SL15 was significantly reduced (by 3.43–6.43%), and fiber length (FL) was remarkably increased (1.1–1.4 mm) (Figures 1A–C), although the other fiber-related traits, namely fiber strength, micronaire and fuzz content not different (Supplementary Figures 2, 3). These results suggest that SL15 has a distinct fiber developmental performance relative to LMY22, and it is suitable as genetic germplasm for further studying fiber development.

### Decreased Single-Fiber Weight Results in a Lower LP in SL15

**Figure 2:**
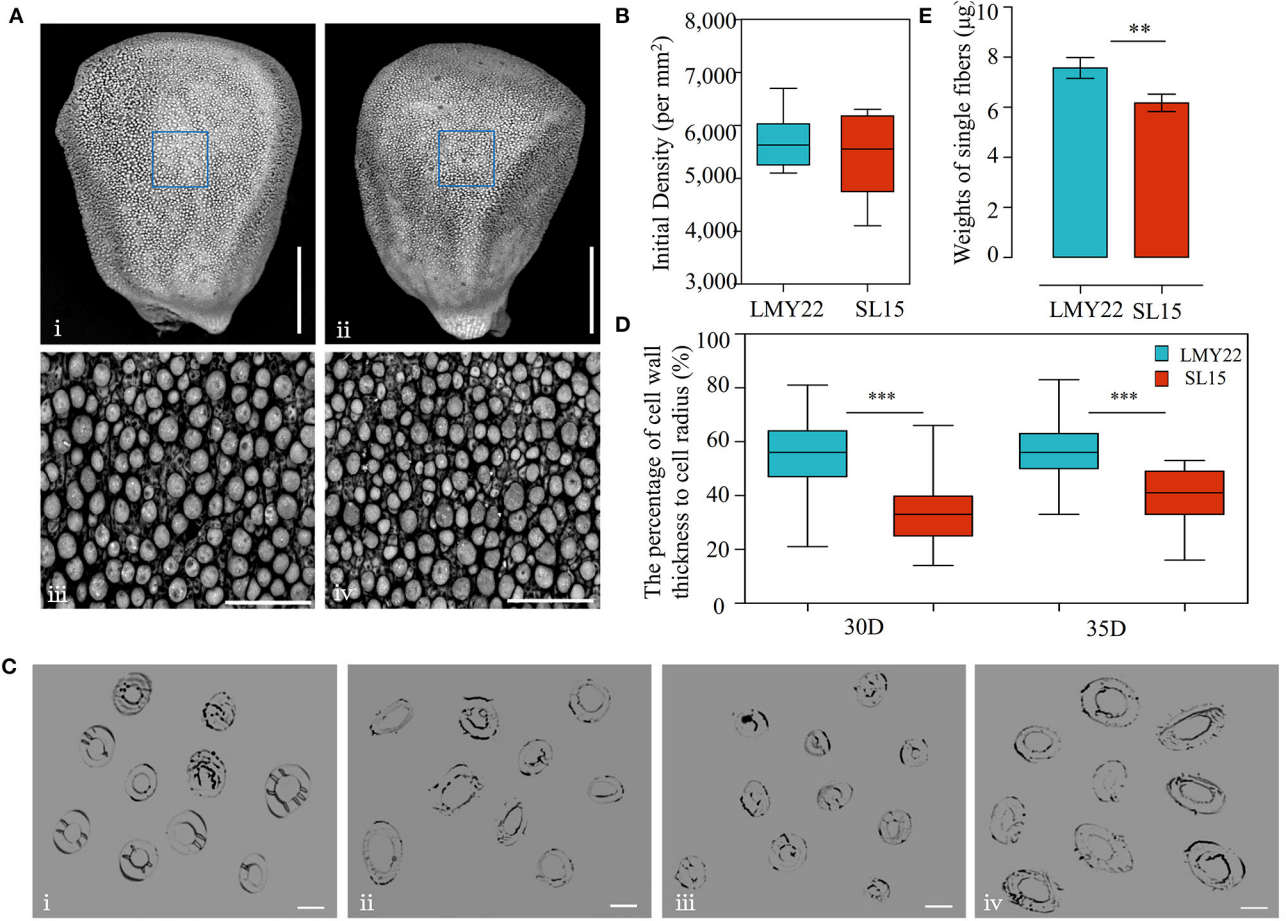
Detection of fiber initiation and cell wall thickness. **(A)** TEM view of fiber initiation at 0 DPA; (i) and (ii) present the whole ovule of LMY22 and SL15, respectively, scale bar, 500 μm; (iii) and (iv) present close-up views of LMY22 and SL15, respectively, scale bar, 50 μm. **(B)** Counts of fiber initiations, mean ± s.d., *n* = 20. **(C)** Cross-sections of cotton fiber cells; (i) and (ii) present LMY22 and SL15 at 30 DPA, respectively; (iii) and (iv) present LMY22 and SL15 at 35 DPA, respectively; scale bar, 20 μm. **(D)** Ratio of cell wall thickness to cell radius (as percentage), mean ± s.d., *n* = 100, ***P < 0.001. **(E)** Weights of single fibers, mean ± s.d., *n* = 50, **P < 0.01 and ***P < 0.001.

LP is an important lint yield component that is affected by two main factors: the fiber numbers on the ovule surface and the single-fiber weight. To explore the factors leading to decreased LP in SL15, the number of fiber protrusions at 0 DPA ovules (lint initiation stage) was investigated by scanning electron microscopy, and no significant difference was observed between SL15 and LMY22 (Figures 2A,B), suggesting that initial lint number did not cause decreased LP in SL15.

Microscopy was used to measure the fiber cell wall thickness at 30 and 35 DPA (Figure 2C), when fiber SCW thickening entered the final stage. The ratio (percentage) of cell wall thickness to cell diameter was calculated, and the mean percentage was 33% in SL15 at 30 DPA, which was much lower than that of LMY22 (55%). The low percentage remained until 35 DPA in SL15 (Figure 2D), indicating that SL15 had a thinner fiber cell wall. This may be an underlying factor leading to decreased LP in SL15.

Interestingly, given an equal weight of dry cotton fibers, the number of lint fibers in SL15 was dramatically increased (Supplementary Figure 4), revealing that its single-fiber weight was significantly decreased (Figure 2E). Taken together, these results indicated that the decreased single-fiber weight resulting from a thinner fiber cell wall may be responsible for the lower LP in SL15.

### The Transcriptome Provides Insights Into the Decreased LP and Elongated Fiber of SL15

**Figure 3:**
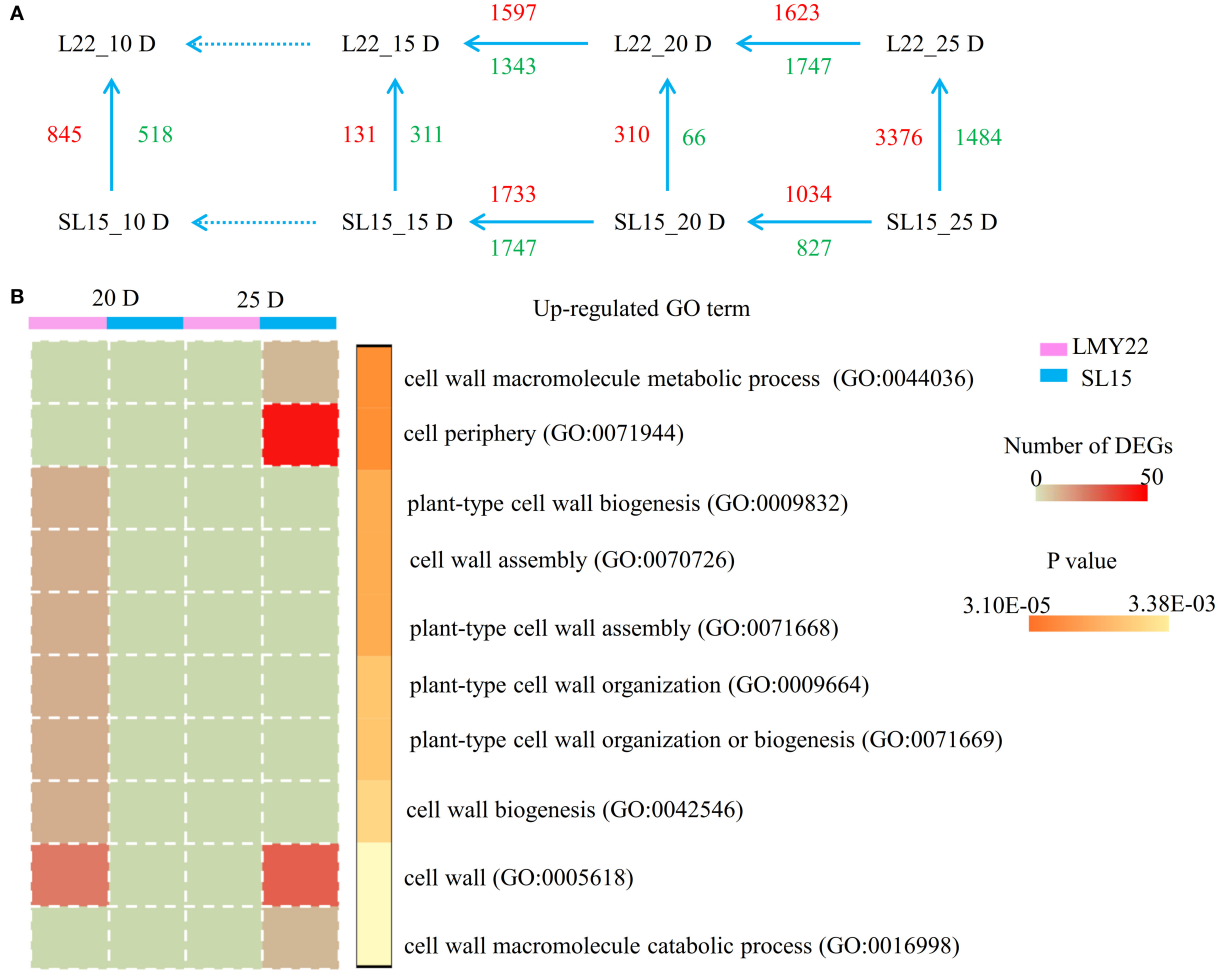
Global transcriptome analysis. **(A)** Numbers of DEGs within and between LMY22 and SL15. **(B)** GO analysis comparing 20 DPA and 25 DPA in LMY22 and SL15.

To identify genes associated with fiber cell development, transcriptional changes were assessed in 10, 15, 20, and 25 DPA fibers of LMY22 and SL15 (Supplementary Figure 5, Supplementary Table 2). Principal component analysis (PCA) revealed that the expression patterns of the two genotypes were similar at any given time point except 20 and 25 DPA (Supplementary Figure 11). Notably, the transcriptome profiles of SL15_25D and LMY22_20D had higher similarity than those of SL15_25D and LMY22_25D (Supplementary Figure 11), which suggested that the fiber development of SL15 at 25 DPA may be similar to that of LMY22 at 20 DPA. Throughout fiber cell development, a total of 6,231 DEGs were identified, and the greatest number of DEGs (4,860) was observed from the comparison of SL15_25D and LMY22_25D (Figure 3A).

To explore the difference in transcriptional regulation between SL15 and LMY22 during fiber SCW development, DEGs were selected at three time points (15, 20, and 25 DPA) for further analysis. Enrichment analysis of DEGs was performed using GO and the KEGG terms (Supplementary Figures 6, 7, Supplementary Tables 3–6). Vertical comparison of DEGs within genotype showed that more up-regulated DEGs involved in cell wall formation occurred at 20 DPA in LMY22, while in SL15, more up-regulated genes were present at 25 DPA (Figure 3B). Additionally, MapMan (Thimm et al., 2004) was used to visualize pathways at 25 DPA, and up-regulated genes in SL15 were enriched in the glycolytic pathway, especially in the break-up of pyruvate into acetaldehyde and lactate (Supplementary Figure 8).

**Figure 4:**
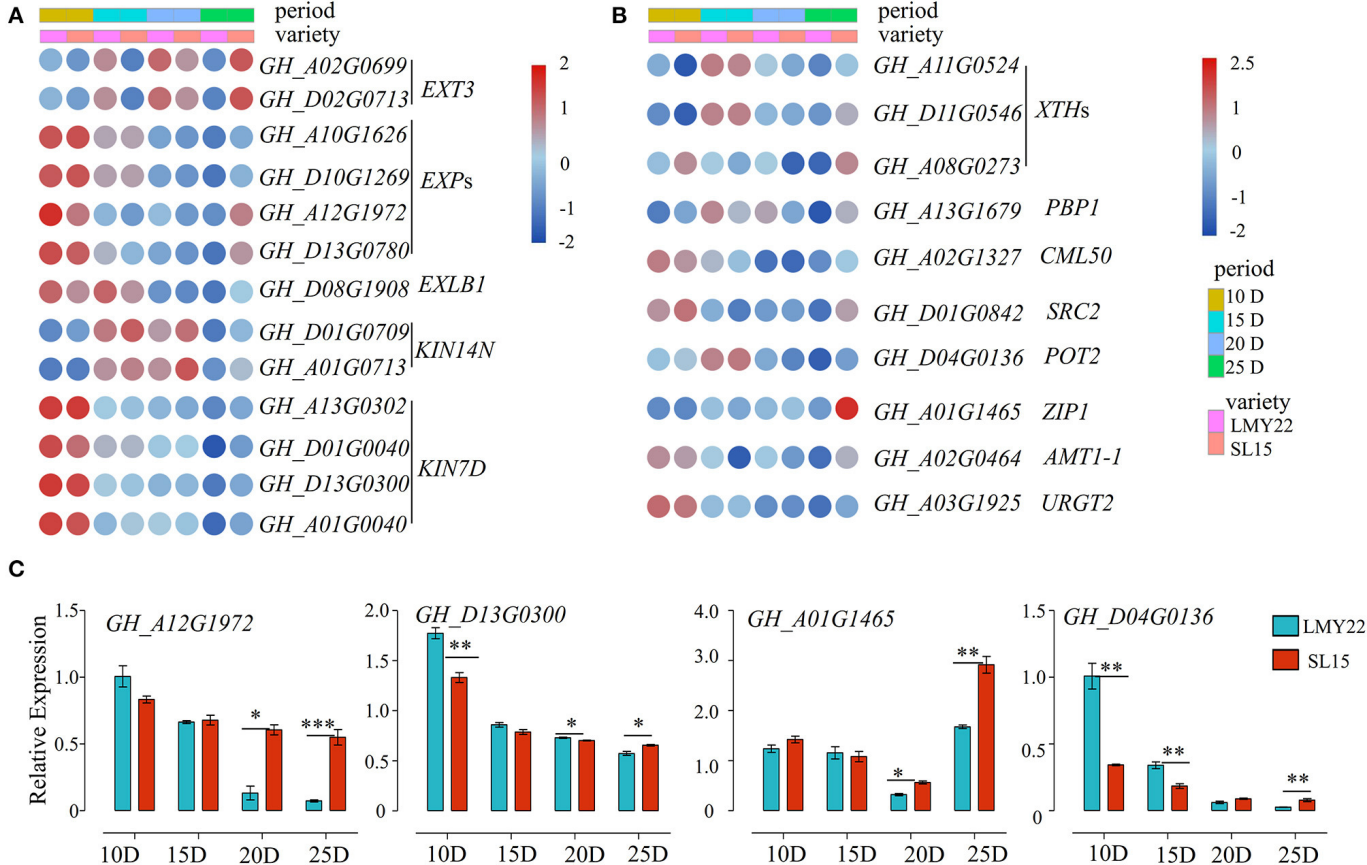
Expression patterns of genes related to fiber elongation. Heat map for DEGs involved in **(A)** cell expansion and **(B)** cell wall loosening. **(C)** Expression of four DEGs as determined by qRT-PCR. *P < 0.05, **P < 0.01, and ***P < 0.001.

Fiber length is principally determined by cotton fiber elongation from 5 to 25 DPA, and the maximum elongation rate was observed at 10 DPA (Li et al., 2017). Cotton fiber elongation is driven by the cell turgor generated by an influx of osmoregulatory solutes together with high expression of genes involved in loosening the cell wall matrix (Ruan and Furbank, 2001; Andres et al., 2014). Notably, the genes encoding extensin, expansin, and kinesin, which are involved in plant cell growth, were highly expressed at 10 DPA (Figure 4A). Furthermore, the expression level of *expansin* (*GhEXP*s) and *kinesin* (*GhKIN7D*) in SL15 declined as fiber maturation progressed, similar to that in LMY22 (Figure 4A). However, the expression of these genes decreased more slowly in SL15 fibers, resulting in their comparative up-regulation at 25 DPA (Figure 4A). The prolonged expression of these genes promotes cell growth and mediated cell wall extension (Cosgrove, 1997; Li et al., 2012), thus potentially promoting fiber elongation by alleviating the limitation of the cell wall. Expression levels of a representative *GhEXP* (*GH_A12G1972*) and *GhKIN7D* (*GH_D13G0300*) were further confirmed by qRT-PCR (Figure 4C).

Plant cell wall extensibility is reportedly mediated by xyloglucan endotransglycosylases/hydrolases (XTHs) (Lee et al., 2010). The expression patterns of *GhXTH*s in our transcriptomes were similar to the aforementioned expansion-related genes (Figure 4B). In addition, three DEGs encoding calcium-binding proteins (*PBP1, CML50*, and *SRC2*) were highly expressed in SL15_10D and SL15_25D (Figure 4B). It is well-known that potassium (K^+^) plays an important role during cotton fiber elongation. *GH_D04G0136*, encoding a potassium transporter, was highly expressed at 15 DPA and then gradually declined; this gene was also up-regulated at 25 DPA fibers in SL15 fibers compared to those of LMY22 (Figure 4B). Similarly, other transporter genes, such as *AMT1-1, ZIP1*, and *URGT2*, were also up-regulated in SL15_25D (Figure 4B). Two transporter genes were randomly selected and confirmed by qRT-PCR (Figure 4C). Together, these results suggest that the fiber cells of SL15 undergo more vigorous cell wall loosening and higher turgor during fiber elongation and SCW thickening stages.

### The Putative NAC-MYB-CESA Network Affects LP by Regulating Fiber SCW Development

**Figure 5:**
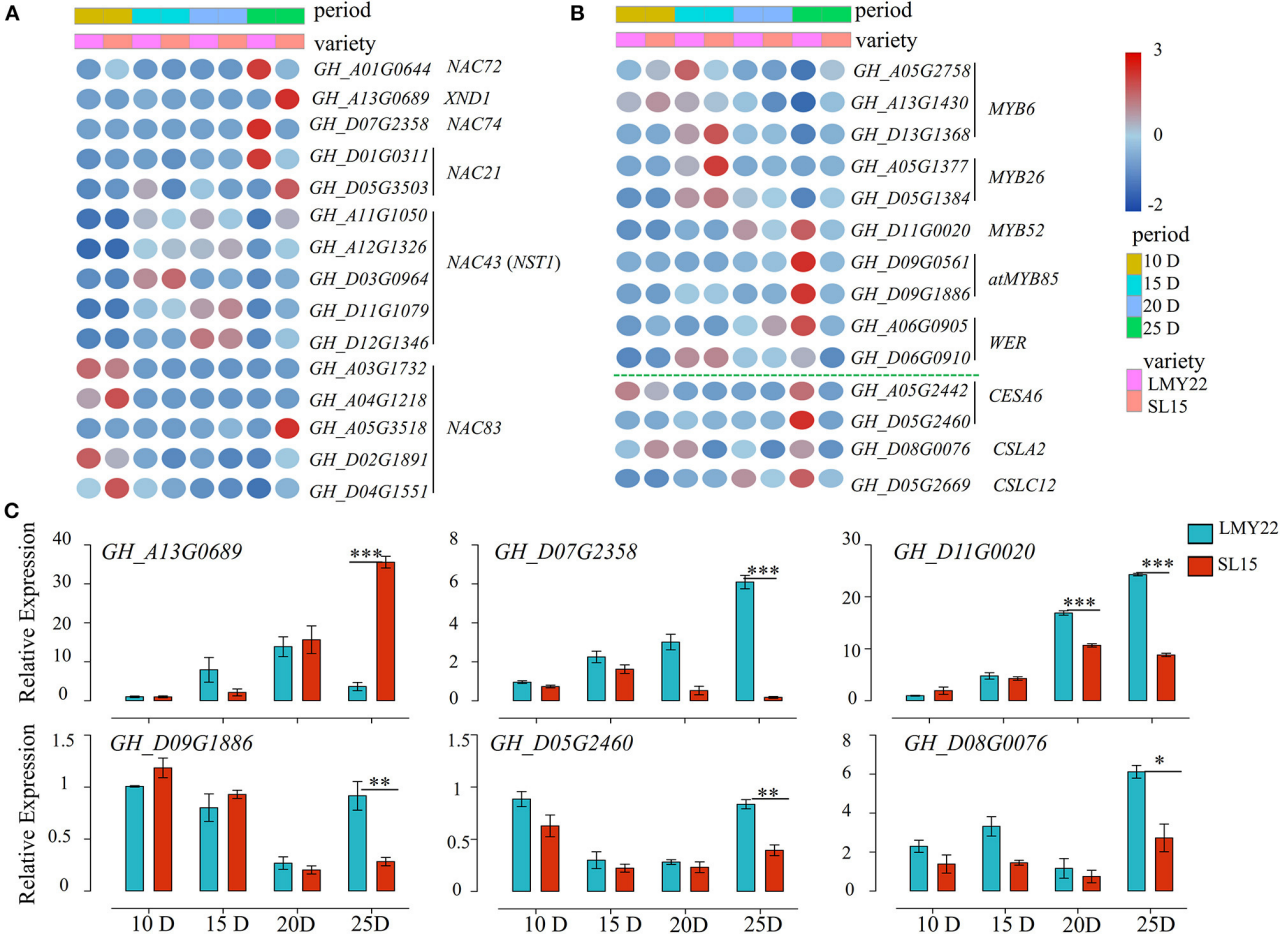
Expression patterns of genes in the NAC-MYB-CESA network. **(A)** Heat map for DEGs encoding NAC transcription factors. **(B)** Heat map for DEGs encoding MYB-like transcription factors and cellulose synthase/synthase-like genes. **(C)** Expression of six DEGs as determined by qRT-PCR. *P < 0.05, **P < 0.01, and ***P < 0.001.

Transcription factors (TFs) play key roles in plant cell wall development (Zhang et al., 2018; Wessels et al., 2019; Sun et al., 2020). In this study, a total of 527 TFs belonging to 32 gene families were identified as involved in cotton fiber development (Supplementary Figure 9), including the MYB, WRKY, and NAC family members. They were simultaneously expressed at four stages. NAC members are known to be critical for plant cell wall development (Wang et al., 2011; Valdivia et al., 2013). Four DEGs (*GH_A03G1732, GH_A04G1218, GH_D02G1891*, and *GH_D04G1551*) were gradually low expression as fiber maturation progressed in both genotypes. However, they were highly expressed at SL15_25D fiber when compared with LMY22_25D (Figure 5A), and *NAC83* (*GH_A05G3518*) had a unique expression pattern that was only highly expressed in SL15_25D (Figure 5A). NST1 (NAC SECONDARY WALL THICKENING PROMOTING FACTOR1) and XND1 (XYLEM NAC DOMAIN 1) have been reported to interact with each other and regulate secondary cell formation in *Arabidopsis thaliana* (Zhang et al., 2019). In our transcriptome, five *NST1*s had similar expression patterns between SL15 and LMY22 at the same time point (Figure 5A). However, the transcriptional level of *XND1* was sharply up-regulated in SL15_25D (Figure 5A). In contrast, the gene encoding NAC72, which is related to senescence (Li et al., 2016), had lower expression in SL15_25D than that in LMY22_25D (Figure 5A). Coincidentally, NAC74 (*GH_A07G2358*) was also down-regulated in SL15_25D; its homologous gene *KIR1* (*AT4G28530*) is known to positively regulate programmed cell death in the stigma of *Arabidopsis* (Gao et al., 2018).

The number of differentially expressed MYB genes was greatest at 25 DPA compared to other time points (Supplementary Figure 9D). Expression pattern analysis showed that *MYB*s involved in the SCW formation pathway were less expressed in SL15_25D than in LMY22_25D. For instance, two *MYB85*s were lowly expressed at all points in SL15, but highly expressed in LMY22 at 25 DPA (Figure 5B). Additionally, *MYB6*s were down-regulated from 15 to 25 DPA were higher in SL15_25D than in LMY22_25D (Figure 5B). In contrast, *GhMYB52* and *GhWER* had increased expression from 15 to 25 DPA in both genotypes, but they had lower expression in SL15_25D compared to LMY22 (Figure 5B).

The DEGs related to cell wall biosynthesis were further investigated, and two cellulose synthase 6 (*CESA6*) genes were gradually increased in LMY22 during fiber SCW thickening stages (Figure 5B). However, the expression of those *CESA6*s was lowly increased in SL15 (Figure 5B); thus, the expression of *CESA6*s was significantly lower in SL15_25D than in LMY22_25D (Figure 5B). Similarly, cellulose-synthase-like C12 (*CSLC12*) was down-regulated in SL15_25D (Figure 5B). Another cellulose-synthase-like gene, *CSLA2*, was also down-regulated in SL15_15D and SL15_25D (Figure 5B). Six DEGs, including *GhXND1* (*GH_A13G0689*), *GhNAC74* (*GH_D07G2358*), *GhMYB*s (*GH_D01G0020* and *GH_D09G1886*), and *GhCESA6* (*GH_D05G2640*), were randomly selected to conduct qRT-PCR and confirmed the reliability of our transcriptome data (Figure 5C). Collectively, these results suggested that the up- or down-regulated expression of genes in the NAC-MYB-CESA network during fiber SCW development may lead to decreased wall thickness and LP in SL15.

### Candidate Genes Associated With LP Formation

**Figure 6:**
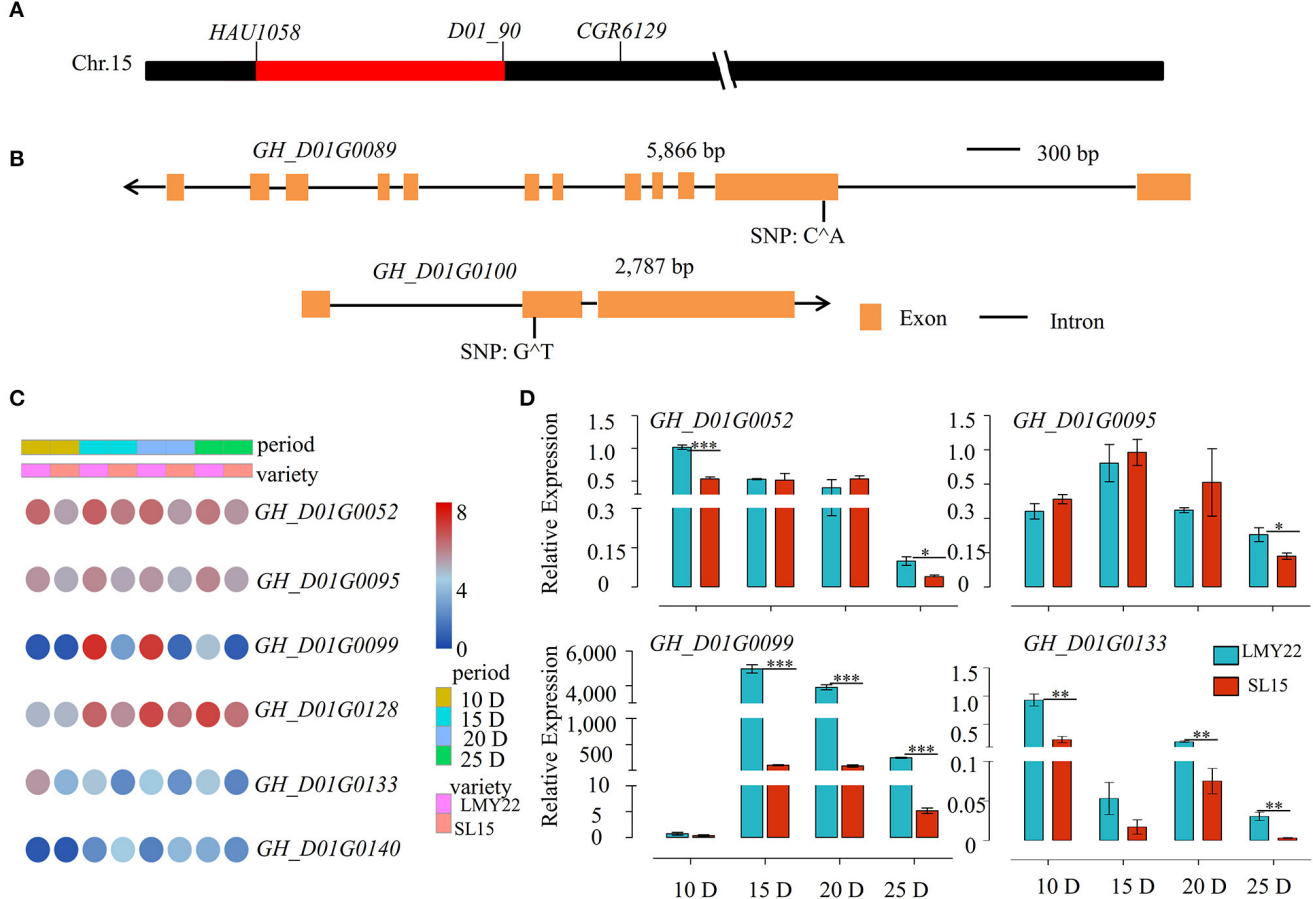
Genetic structural variation and Expression patterns of candidate genes. **(A)** A schematic of introgressive segment on Chromosome 15 of SL15. **(B)** The genetic structural variation of candidate genes. Orange yellow boxes represent exons and black lines represent introns; the arrow represents the direction of genes on the chromosome. **(C)** Expression heat map for six candidate DEGs. **(D)** Expression of candidate genes as determined by qRT-PCR. *P < 0.05, **P < 0.01, and ***P < 0.001.

SL15 was developed due to a major QTL for LP (*qLP-C15-1*) detected in our previous study (Wang et al., 2020). Based on the genetic map (Supplementary Figure 1), the substituted chromosome 15 segment interval was further determined in SL15 *via* increasing marker density to identify the candidate genes associated with lint-percentage. The substituted segment was finally narrowed down to a 1069-Kbp genomic region flanked by two SSR markers, *HAU1058* and *D01_90* (Figure 6A). According to TM-1 Refseq v2.0 sequences, this region contains 146 candidate genes (Supplementary Table 7). The genetic variation of these candidate genes was investigated using DNA resequencing data from LMY22 and LY343 (the donor parent of SL15), and one non-sense mutation each was observed in the exon of *GH_D01G0089* (Exon2: c.C382A; p.E128^*^) and *GH_D01G0100* (Exon2: c.229T; p.E77^*^), leading to premature translation termination (Figure 6B).

The transcriptome profile of all candidate genes was further examined within the interval, and 96 genes were FPKM>1 and expressed at all fiber development time points, in which six genes were differentially expressed in all four periods (Figure 6C). Among those, *GH_D01G0052, GH_D01G0095*, and *GH_D01G0128* were highly expressed during all fiber cell development and were correspondingly lower in SL15 than in LMY22; *GH_D01G0133* was more highly expressed in fibers at 10 DPA than the other periods, while *GH_D01G0099* and *GH_D01G0140* had almost no expression at 10 DPA. *GH_D01G0099* showed a sharp increased expression at 15 DPA, followed by a gentle decrease at 20 DPA and an abrupt decline at 25 DPA (Figure 6C). Four differently expressed candidate genes in this QTL region were randomly selected and confirmed by qRT-PCR (Figure 6D). The SNP or Indel variation in the promoter region may cause the differential expression of the candidate genes. Therefore, promoter variation of these six DEGs was investigated using our resequencing data. The results revealed that many SNP mutations were detected in the promoter region (Supplementary Table 8).

To identify candidate genes co-participating in LP formation, the cluster R package was used to analyze the expression patterns of all DEGs, and eight clusters were identified in SL15 (Supplementary Figure 10). Most of the candidate genes were grouped into clusters 1 and 2 (Supplementary Figure 10). Notably, candidate genes *GH_D01G0052, GH_D01G0099, GH_D01G0100*, and *GH_D01G0140* together with *CESA6* and *COBRA-like* were co-expressed in cluster 1 (Supplementary Figure 10, Supplementary Table 9). These results suggest that *GH_D01G0052, GH_D01G0099, GH_D01G0100*, and *GH_D01G0140* may be the core candidate genes associated with *qLP-C15-1*.

## Discussion

### Reduction of Cell Wall Thickness Is the Major Cause for Lower LP in SL15

In our previous study, a substitution line SL15, which substituted a LY343 chromosomal segment containing an LP QTL (*qLP-C15-1*) into the LMY22 genetic background, was developed, leading to a reduction in LP, as observed in field trials conducted over consecutive years. There was no significant difference in the boll weight, seed index, and fuzz content between SL15 and LMY22. Therefore, SL15 can be regarded as a mutant germplasm to mine the core genes regulating LP formation.

Currently, germplasms with low LP mostly result from a reduction in the initial fiber number (Ma et al., 2016; Hu et al., 2018a). In contrast, our present anatomical images revealed that the lower LP in SL15 mainly resulted from reduced thickness of fiber cell walls. Few genes have been identified to affect LP through involvement in fiber SCW development. Thus, SL15 and LMY22 are ideal near-isogenic lines to identify key genes regulating LP formation and provide a new insight into the cellular and molecular biological mechanisms that determine LP, the main cotton lint yield component.

### Multiple Pathways Affect Fiber Elongation and SCW Thickening

Genome-wide transcriptome profiling can be used effectively to uncover transcriptional regulatory mechanisms that impact plant development and growth (Yoo and Wendel, 2014; Islam et al., 2016; Garg et al., 2017; Wang et al., 2018). In a previous study, the transcriptome of the high fiber strength germplasm SL7 was dissected, and its introgressed chromosomal segment regulated a hormone-transcription factor hierarchical regulatory network that induces the expression of SCW-related genes (Song et al., 2020). The present study compared the transcriptomes of a CSSL (SL15) and its recurrent parent (LMY22) during fiber cell development and identified a total of 6,231 DEGs. Within-genotype GO analysis of DEGs from the adjacent periods of 20 and 25 DPA revealed that upregulated DEGs of LMY22_20D were enriched in cell-wall-related GO terms (Supplementary Table 10). Five of these DEGs encoded a member of the COBRA-like extracellular glycosyl-phosphatidyl inositol-anchored protein family, homologous to the IRX6 (*AT5G15630*) protein in *Arabidopsis*; the putative cotton homolog, *GhCOBL9A*, was responsible for plant cell elongation and thickening (Niu et al., 2018). However, differential expression of this gene was not detected in SL15 (Supplementary Table 11). Finally, several DEGs upregulated in SL15_25D that annotated with the GO terms “cell periphery” (GO: 0071944) and “cell wall” (GO: 0005618) were detected in LMY22_20D (Figure 3B, Supplementary Table 11). Consequently, development of the cotton fiber SCW was not synchronous for SL15 and LMY22.

SCW is primarily composed of cellulose, which is in turn composed of UDP-glucose-bound substrates supplied by sucrose synthase or UDP-Glc pyrophosphorylase through carbon partitioning (Haigler et al., 2001; Verbančič et al., 2018). Using MapMan pathways, SL15_25D was found to feature many more upregulated DEGs in sucrose glycolysis, especially the pyruvate decomposition pathway. This abnormal carbohydrate metabolism may affect cellulose biogenesis. A high content of osmotically active solutes has been reported as a major factor in the imposition of maximal turgor pressure for fiber elongation (Hu et al., 2019). The upregulated DEGs included lactate dehydrogenase and pyruvate decarboxylase, which may generate many metabolites that act as osmoregulatory solutes to change or increase vacuole turgor pressure, thereby driving fiber elongation. Meanwhile, DEGs associated with cell expansion and cell wall loosening were also highly expressed in SL15_25D. Furthermore, K^+^ transporter genes were also upregulated in SL15_25D, providing abundant potassium to maintain cell turgor pressure for fiber elongation (Guo et al., 2017).

Transcription factors (TFs) also act as critical regulators of secondary cell wall biosynthesis. Cotton fiber is developed from a single ovule epidermal cell, so the development process of fiber is almost similar to plant cells. Based on the NAC-MYB-CESA network involved in cell wall biosynthesis in the model plant *Arabidopsis thaliana* (Zhong et al., 2008, 2010; Wang and Dixon, 2012), the DEGs related to the NAC-MYB-CESA network were investigated, and some of them were up- or down-regulation, affecting fiber SCW thickening of SL15. NAC83 (*AT5G13180*) has been reported to interact with VASCULAR-RELATED NAC-DOMAIN7 (VND7) to negatively regulate xylem vessel formation in *Arabidopsis* (Yamaguchi et al., 2010). In this study, differentially expressed transcripts encoding NAC83 were upregulated in SL15_25D and may negatively regulate fiber SCW development. *NST1* is a key gene that initiates SCW formation through a hierarchical transcription network; its transcriptional activity is inhibited by XND1 (Zhang et al., 2019). Here, DEGs encoding XND1 were sharply upregulated in SL15_25D, suggesting full repression of NST1 transcriptional activity at that time point. Additionally, the greatest number of DEGs encoding MYB family members was observed at 25 DPA. MYB6 has been reported to reduce secondary cell wall deposition through interacting with KNAT7 in poplar and *Arabidopsis* (Wang L. et al., 2019); its homologous gene *GH_A05G2758* was upregulated in SL15_25D. The expression of these transcriptional regulators eventually leads to a decline in the expression of *CESA6, CSLC12*, and *CSLA2* in SL15. Overexpression of the *AtCesA6-like* genes was also responsible for increased secondary cell wall deposition, and led to improved mechanical strength and higher biomass production in transgenic *Arabidopsis* (Hu et al., 2018b). Those DEGs were up- or downregulated in the putative NAC-MYB-CESA network and affected the fiber SCW thickness of SL15. In contrast, the NAC TFs *GhNAC72* (Li et al., 2016) and *GhNAC74* (Gao et al., 2018), involved in the positive regulation of plant senescence and programmed cell death, respectively, were also downregulated in SL15, maintaining cell life activities to prolong the period of fiber cell elongation.

### Candidate Genes Located in the Substituted Fragment of SL15

Numerous candidate genes involved in regulating LP have been identified to date (Su et al., 2016; Han et al., 2020; Wang et al., 2020). Here, candidate genes were mined in the introgression fragment of a major QTL for LP by resequencing data and transcriptome analysis. The expression of *GH_D01G0089* and *GH_D01G0100* was not significantly changed between SL15 and LMY22 during fiber development, but stop-gain SNP mutations were observed in the exon. The candidate gene *GH_D01G0089* encoded a vacuolar sorting receptor 3 protein and was homologous to ATVSR3 (*AT2G14740*), playing an important role in response to plant stress (Avila et al., 2008). *GH_D01G0100* encoded a polyol/cyclitol/monosaccharide-H^+^-symporter and was homologous to ATPMT5 (*AT3G18830*), which is involved in plant cell wall modifications (Klepek et al., 2009).

In addition, four of six differently expressed candidate genes, namely *GH_D01G0052, GH_D01G0095, GH_D01G0128*, and *GH_D01G0154*, were differentially expressed in each period of fiber development. *GH_D01G0052* and *GH_D01G0095* encoded a transmembrane protein (TMN1) and a magnesium transporter (MGT4), respectively, acting as mediators of the cellular content of metals (Cu and Mg, respectively) and being involved in plant cell development (Hegelund et al., 2010; Li J. et al., 2015). A distinctive expression pattern was observed for *GH_D01G0099*, which was initially expressed at 15 DPA and peaked at this point. The expression of *GH_D01G0099* in the fiber cell transition and SCW thickening period of SL15 was dozens of times lower than that of LMY22. Its *Arabidopsis* homolog (*AT1G47530*) was reported to be widely expressed in all plant tissues, and as a turgor-regulating chloride channel, it is involved in various plant life activities (Zhang H. et al., 2017). Co-expression analysis showed that four candidate genes, *GH_D01G0052, GH_D01G0099, GH_D01G0100*, and *GH_D01G0140*, shared expression patterns with *CESA6* and *COBRA-like* (cluster 1), which may co-participate in an unknown pathway to affect secondary cell wall thickening.

**Figure 7:**
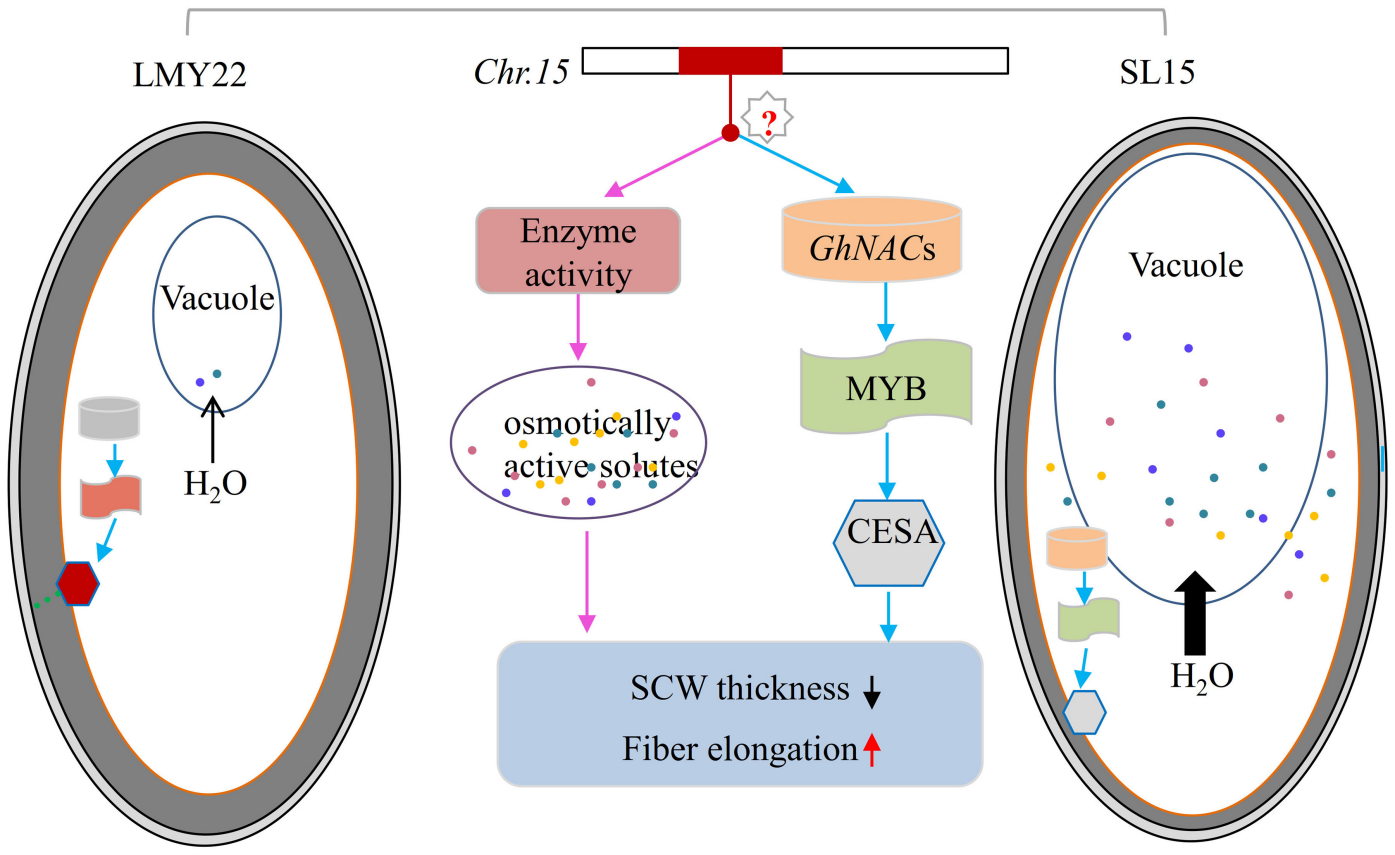
Model of fiber secondary cell wall thickening in LMY22 and SL15. Light gray and dark gray ovals respectively represent the cotton fiber primary cell wall and secondary cell wall (SCW); the orange oval represents the plasmalemma of the cotton fiber cell. Cylinders represent GhNACs that negatively regulate fiber SCW development, with orange and light gray indicating DEGs with up- or downregulated expression, respectively. Oblong waves represent MYBs involved in cellulose biosynthesis, with brick red and light green indicating DEGs with up- or downregulated expression, respectively. Hexagons represent cellulose synthase or cellulose-synthase-like genes, with bright red and light gray indicating DEGs with up- or downregulated expression, respectively. Colored dots represent various osmotically active solutes. Black arrows qualitatively represent the amount of water flowing from cytoplasm into vacuole.

It was reported that *G. barbadense* has extra-long fibers due to substantially increased osmotically active solutes and prolonged expression of genes involved in fiber elongation (Hu et al., 2019). In this study, transcriptional temporal and spatial differences during fiber development between SL15 and LMY22 were mainly caused by the substituted segment from LY343. Of the chromosome 15 genomic sequence of LY343, 20.66% was obtained from *G. hirsutum* race (Wang et al., 2020), which may be inherited from *G. barbadense* during the early evolutionary events (Fang et al., 2017a). Based on the genetic background of SL15 and the transcriptome data analysis, a model was proposed for *qLP-C15-1* to regulate fiber development (Figure 7). The candidate genes in the substituted fragment controlling fiber elongation and SCW development, as well as LP by regulating the expression of pyruvate catabolase genes to produce more or less metabolites following the change in fiber intracellular osmotic pressure, and expression level of TFs involved in SCW development. However, how the candidate genes in the introgressive segment simultaneously regulate fiber length and SCW thickness should be further explored.

## Data Availability Statement

The datasets presented in this study can be found in online repositories. The names of the repository/repositories and accession number(s) can be found at: NCBI [accession: PRJNA718400].

## Author Contributions

JuZ, TZ, and FW designed the experiments. YG, YC, ZS, WL, HZ, and XH performed field trails, phenotypic evaluation, and data collection. JiZ, LZ, and YG prepared the fiber RNA sample and performed the analysis of RNA-seq data. YG and YC observed fiber phenotype and determined the single fiber weight. YG drafted the manuscript. FW, JuZ, and TZ revised the manuscript. All authors read and approved the final manuscript.

## Funding

This work was financially supported by the National Science Foundation in China (32072116, 31601345); the earmarked fund for China Agriculture Research System (CARS-15-05); the Fundamental Research Funds for the Central Universities (2020XZZX004-03); Leading Innovative and Entrepreneur Team Introduction Program of Zhejiang (2019R01002); Seed-Industrialized Development Program in Shandong Province (2020LZGC002), and the Taishan Scholars Program of Shandong Province (ts201511070).

## Conflict of Interest

The authors declare that the research was conducted in the absence of any commercial or financial relationships that could be construed as a potential conflict of interest.

## Publisher's Note

All claims expressed in this article are solely those of the authors and do not necessarily represent those of their affiliated organizations, or those of the publisher, the editors and the reviewers. Any product that may be evaluated in this article, or claim that may be made by its manufacturer, is not guaranteed or endorsed by the publisher.

## Supplementary Material

Supplementary Figure 1 Identification of the substituted segment in the cotton genome using SSR markers.

Supplementary Figure 2 Comparison of fiber quality and boll weight over multiple years. **(A)** Fiber strength, **(B)** micronaire, and **(C)** boll weight of LMY22 and SL15 based on a four-year field trial from 2016 to 2019 (mean ± s.d., *n* = 5, ^*^*P* < 0.05).

Supplementary Figure 3 Comparison of seed index and seed number of per boll as well as fuzz content. **(A)** Seed index of LMY22 and SL15 in 2018 and 2019 (mean ± s.d., *n* = 50). **(B)** Number of seeds per boll (mean ± s.d., *n* = 20). **(C)** Fuzz content of LMY22 and SL15 (mean ± s.d., *n* = 50).

Supplementary Figure 4 The number of mature fibers per unit weight. **(A)** Fiber number of per unit length (N1), mean ± s.d., *n* = 50, ^**^*P* < 0.01. **(B)** Mean fiber length (mm) was determined by AFIS (mean ± s.d., *n* = 5). **(C)** Number of mature fibers (N2) in 2 mg was calculated from the following equation: (N2) = (N1 ×10)/(the mean fiber length of LMY22 and SL15, respectively), mean ± s.d., *n* = 50, ^**^*P* < 0.01.

Supplementary Figure 5 Number of expressed genes.

Supplementary Figure 6 GO term enrichment analysis of genes differentially expressed between SL15 and LMY22 at **(A)** 15 DPA, **(B)** 20 DPA, and **(C)** 25 DPA.

Supplementary Figure 7 KEGG pathway enrichment analysis of genes differentially expressed between SL15 and LMY22 at **(A)** 15 DPA, **(B)** 20 DPA, and **(C)** 25 DPA.

Supplementary Figure 8 DEGs enriched in the plant glycolytic pathway in SL15_25D.

Supplementary Figure 9 Number of DEGs in common TF families that showed up- or downregulation between SL15 and LMY22 during fiber development: **(A)** 10 DPA, **(B)** 15 DPA, **(C)** 20 DPA, and **(D)** 25 DPA.

Supplementary Figure 10 Expression pattern of candidate genes in SL15 during different fiber development periods. Blue and black numbers respectively count genes in the substituted chromosome segment and DEGs in each cluster.

Supplementary Figure 11 Transcriptome correlation and clustering analyses. **(A)** Spearman correlation coefficient (SCC) analysis of LMY22 and SL15 transcriptomes across fiber development periods. **(B)** Principal component analysis (PCA) plot showing clustering in LMY22 and SL15 transcriptomes during four fiber development periods.

Supplementary Table 1 List of primers used in qRT-PCR verification.

Supplementary Table 2 Summary of transcriptome data for all 16 samples.

Supplementary Table 3 GO annotations for genes differentially-expressed between LMY22 and SL15 at 15 DPA.

Supplementary Table 4 GO annotations for genes differentially-expressed between LMY22 and SL15 at 20 DPA.

Supplementary Table 5 GO annotations for genes differentially-expressed between LMY22 and SL15 at 25 DPA.

Supplementary Table 6 KEGG annotations for genes differentially-expressed between LMY22 and SL15.

Supplementary Table 7 Genes in the chromosomal segment substituted in SL15.

Supplementary Table 8 Information of promoter region SNP mutations of candidate genes.

Supplementary Table 9 Summary of the eight SL15 DEG clusters.

Supplementary Table 10 Genes up-regulated in LMY22_20D and having cell-wall-related GO term annotations.

Supplementary Table 11 Genes up-regulated in SL15_25D and having cell-wall-related GO term annotations.
