# Corrigendum: Comparative Dynamic Transcriptome Reveals the Delayed Secondary-Cell-Wall Thickening Results in Altered Lint Percentage and Fiber Elongation in a Chromosomal Segment Substitution Line of Cotton (*Gossypium hirsutum* L.)

**DOI:** 10.3389/fpls.2022.837797

**Published:** 2022-01-26

**Authors:** Yang Gao, Yu Chen, Zhangqiang Song, Jingxia Zhang, Wanyu Lv, Han Zhao, Xuehan Huo, Ling Zheng, Furong Wang, Jun Zhang, Tianzhen Zhang

**Affiliations:** ^1^State Key Laboratory of Crop Genetics and Germplasm Enhancement, Nanjing Agricultural University, Nanjing, China; ^2^Key Laboratory of Cotton Breeding and Cultivation in Huang-Huai-Hai Plain, Ministry of Agriculture and Rural Affairs, Cotton Research Center, Shandong Academy of Agricultural Sciences, Jinan, China; ^3^College of Life Sciences, Shandong Normal University, Jinan, China; ^4^Zhejiang Provincial Key Laboratory of Crop Genetic Resources, Institute of Crop Science, College of Agriculture and Biotechnology, Plant Precision Breeding Academy, Zhejiang University, Hangzhou, China

**Keywords:** lint percentage, single chromosomal segment substitution line, transcriptome analysis, secondary-cell-wall thickening, candidate genes

In the published article, there was an error in the order of correspondence. Instead of “Jun Zhang/ zj0928@126.com, Tianzhen Zhang/ cotton@njau.edu.cn,” it should be “Tianzhen Zhang/ cotton@njau.edu.cn, Jun Zhang/ zj0928@126.com”

The authors apologize for this error and state that this does not change the scientific conclusions of the article in any way. The original article has been updated.

## Publisher's Note

All claims expressed in this article are solely those of the authors and do not necessarily represent those of their affiliated organizations, or those of the publisher, the editors and the reviewers. Any product that may be evaluated in this article, or claim that may be made by its manufacturer, is not guaranteed or endorsed by the publisher.

