# Supplementary figures and images for: Comparative Dynamic Transcriptome Reveals the Delayed Secondary-Cell-Wall Thickening Results in Altered Lint Percentage and Fiber Elongation in a Chromosomal Segment Substitution Line of Cotton (Gossypium hirsutum L.)
Source: Front Plant Sci. 2021 Oct 25;12:756434. doi: 10.3389/fpls.2021.756434 (PMC8573213; doi:10.3389/fpls.2021.756434)

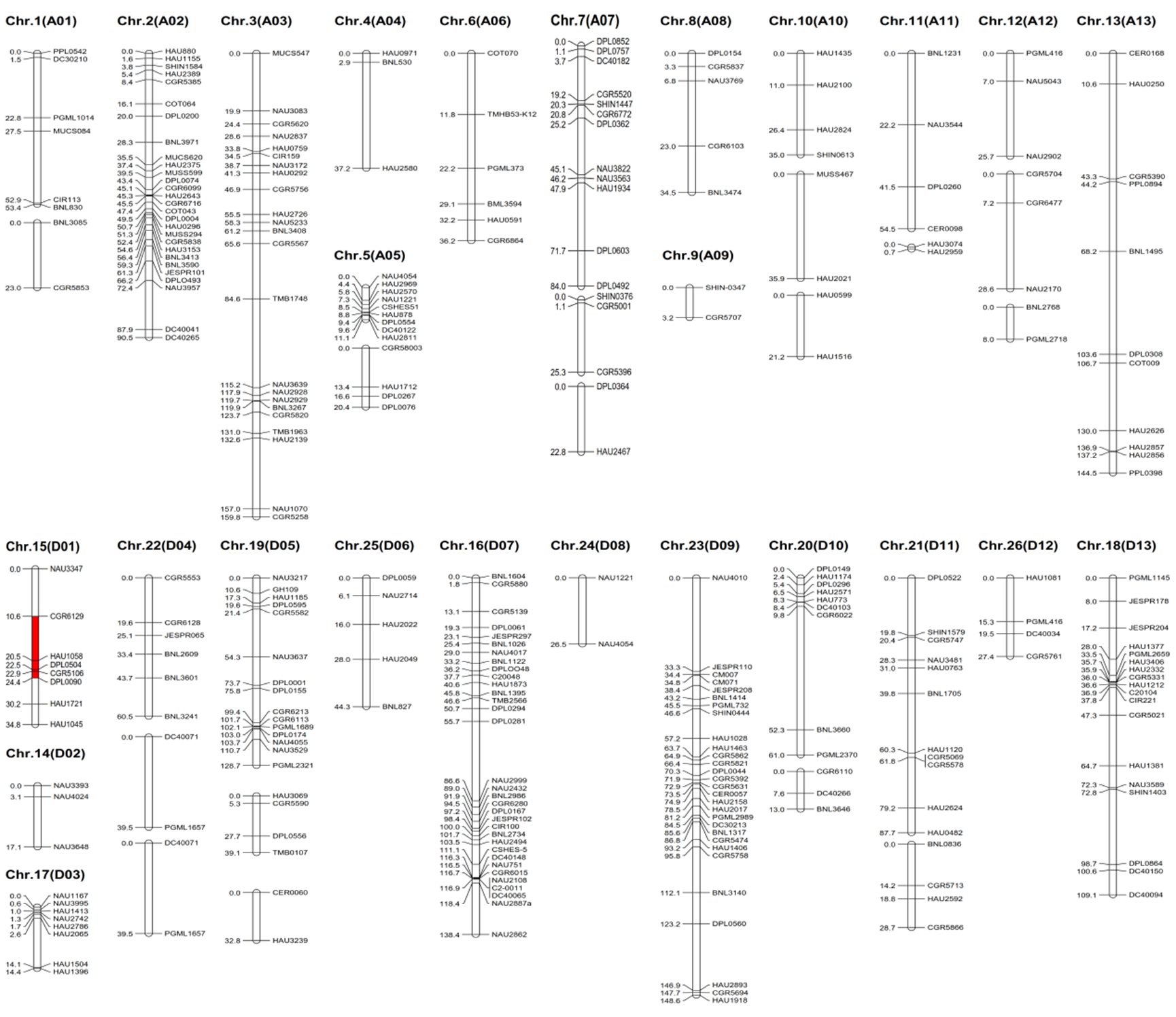

Supplement: Supplementary Figure 1 — Identification of the substituted segment in the cotton genome using SSR markers. [file Image_1.TIF]

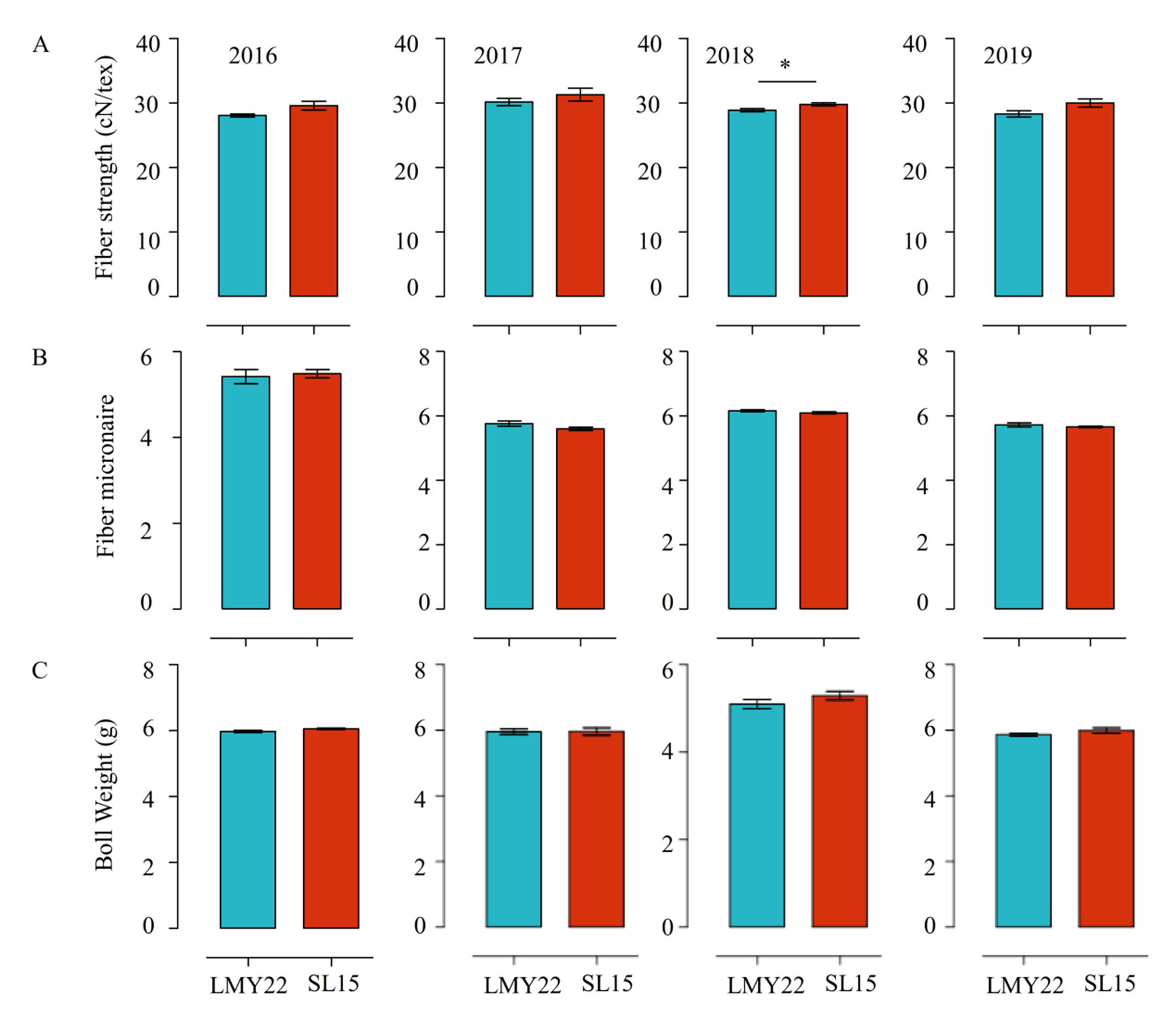

Supplement: Supplementary Figure 2 — Comparison of fiber quality and boll weight over multiple years. (A) Fiber strength, (B) micronaire, and (C) boll weight of LMY22 and SL15 based on a four-year field trial from 2016 to 2019 (mean ± s.d., n = 5, *P < 0.05). [file Image_2.TIF]

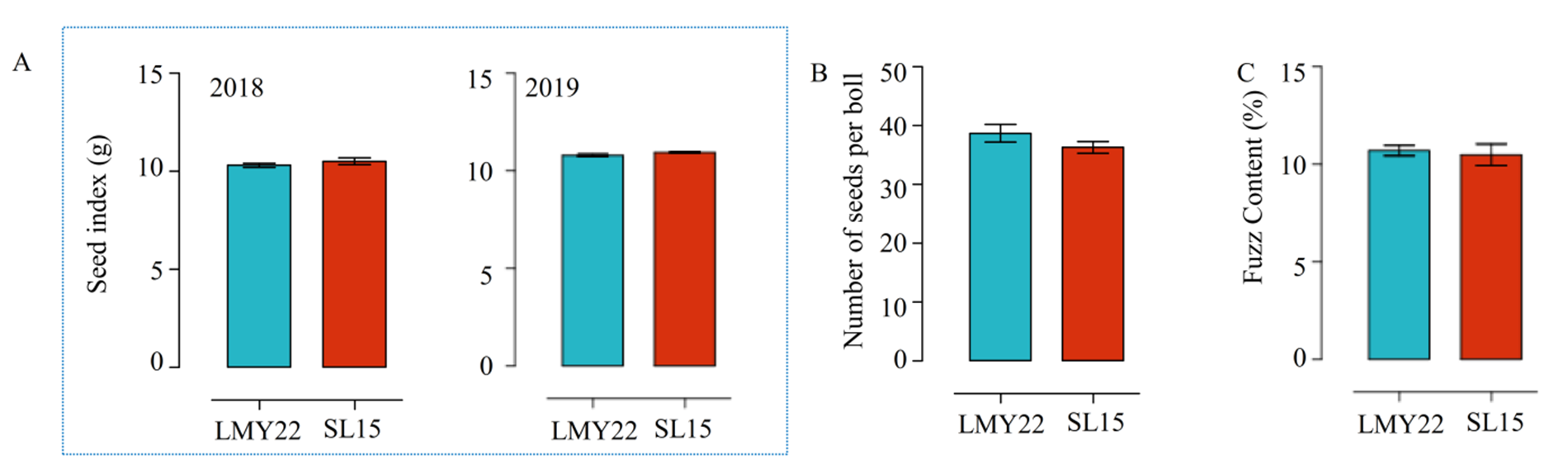

Supplement: Supplementary Figure 3 — Comparison of seed index and seed number of per boll as well as fuzz content. (A) Seed index of LMY22 and SL15 in 2018 and 2019 (mean ± s.d., n = 50). (B) Number of seeds per boll (mean ± s.d., n = 20). (C) Fuzz content of LMY22 and SL15 (mean ± s.d., n = 50). [file Image_3.TIF]

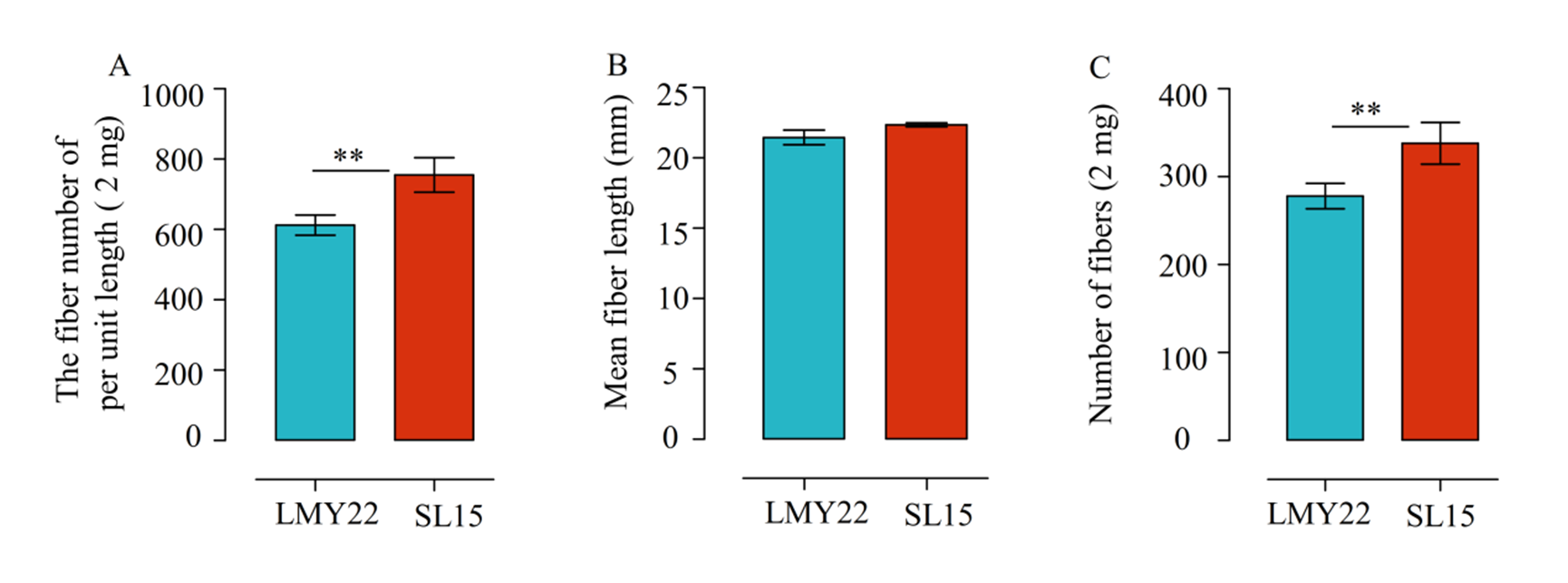

Supplement: Supplementary Figure 4 — The number of mature fibers per unit weight. (A) Fiber number of per unit length (N1), mean ± s.d., n = 50, **P < 0.01. (B) Mean fiber length (mm) was determined by AFIS (mean ± s.d., n = 5). (C) Number of mature fibers (N2) in 2 mg was calculated from the following equation: (N2) = (N1 ×10)/(the mean fiber length of LMY22 and SL15, respectively), mean ± s.d., n = 50, **P < 0.01. [file Image_4.TIF]

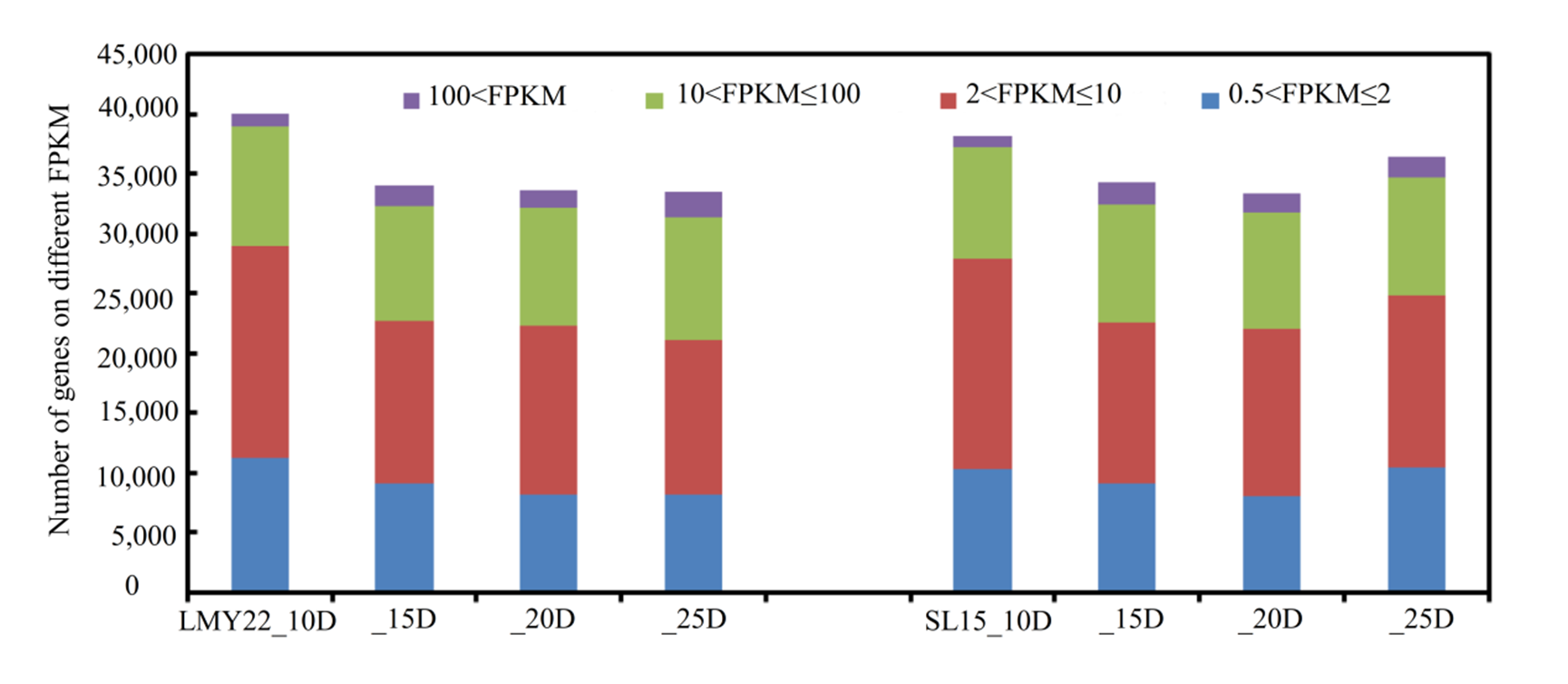

Supplement: Supplementary Figure 5 — Number of expressed genes. [file Image_5.TIF]

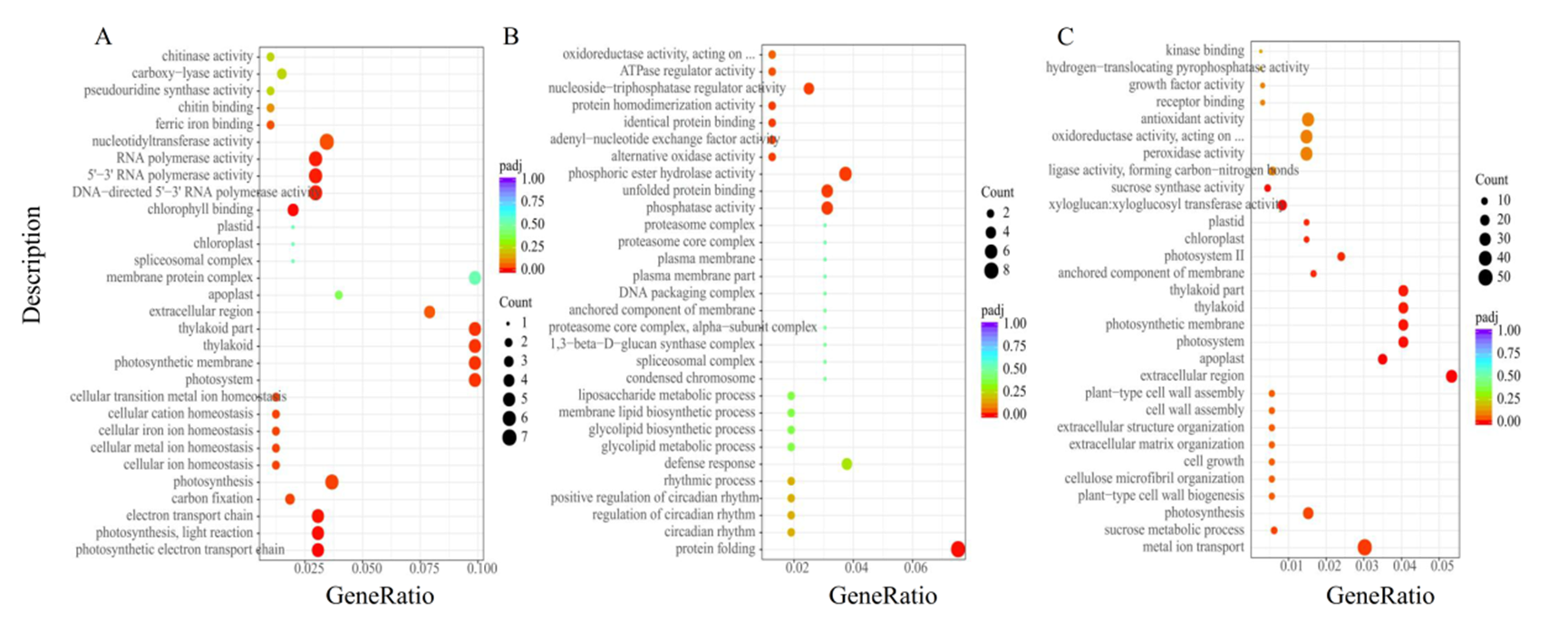

Supplement: Supplementary Figure 6 — GO term enrichment analysis of genes differentially expressed between SL15 and LMY22 at (A) 15 DPA, (B) 20 DPA, and (C) 25 DPA. [file Image_6.TIF]

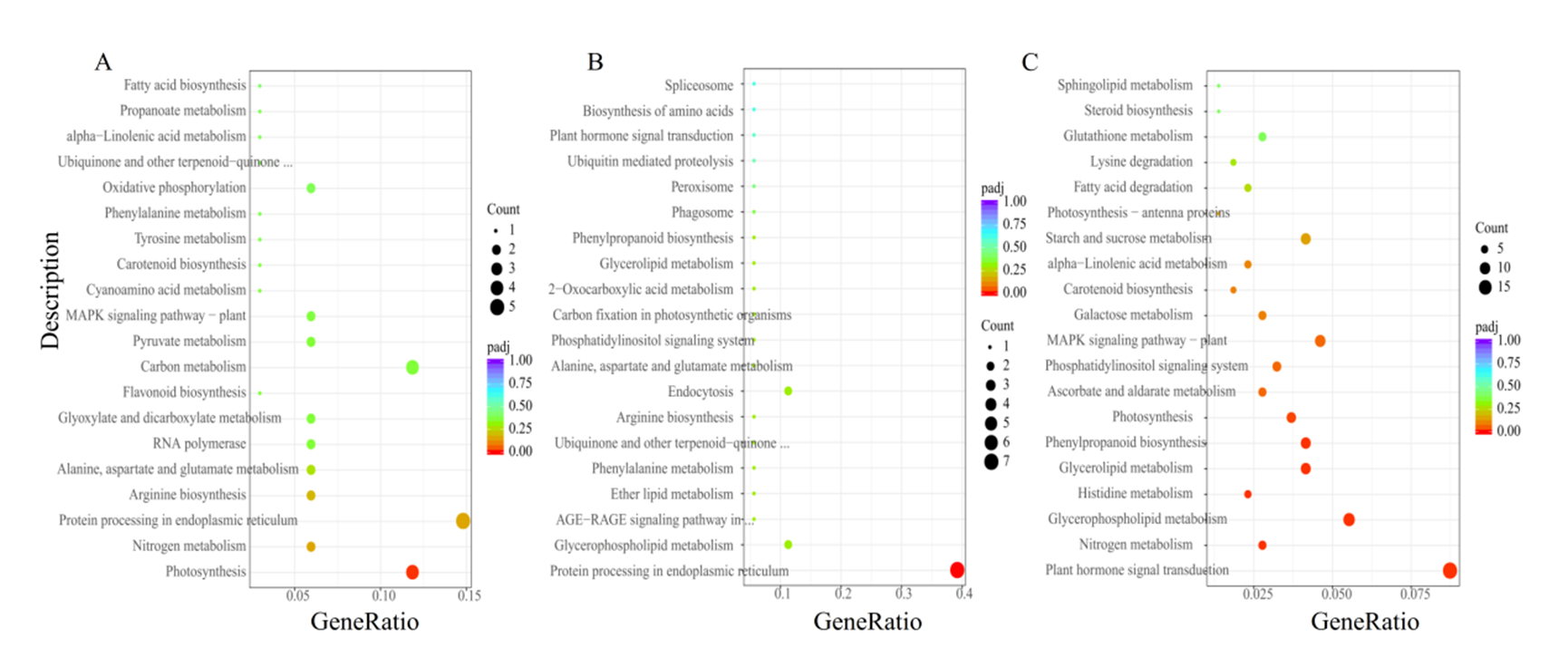

Supplement: Supplementary Figure 7 — KEGG pathway enrichment analysis of genes differentially expressed between SL15 and LMY22 at (A) 15 DPA, (B) 20 DPA, and (C) 25 DPA. [file Image_7.TIF]

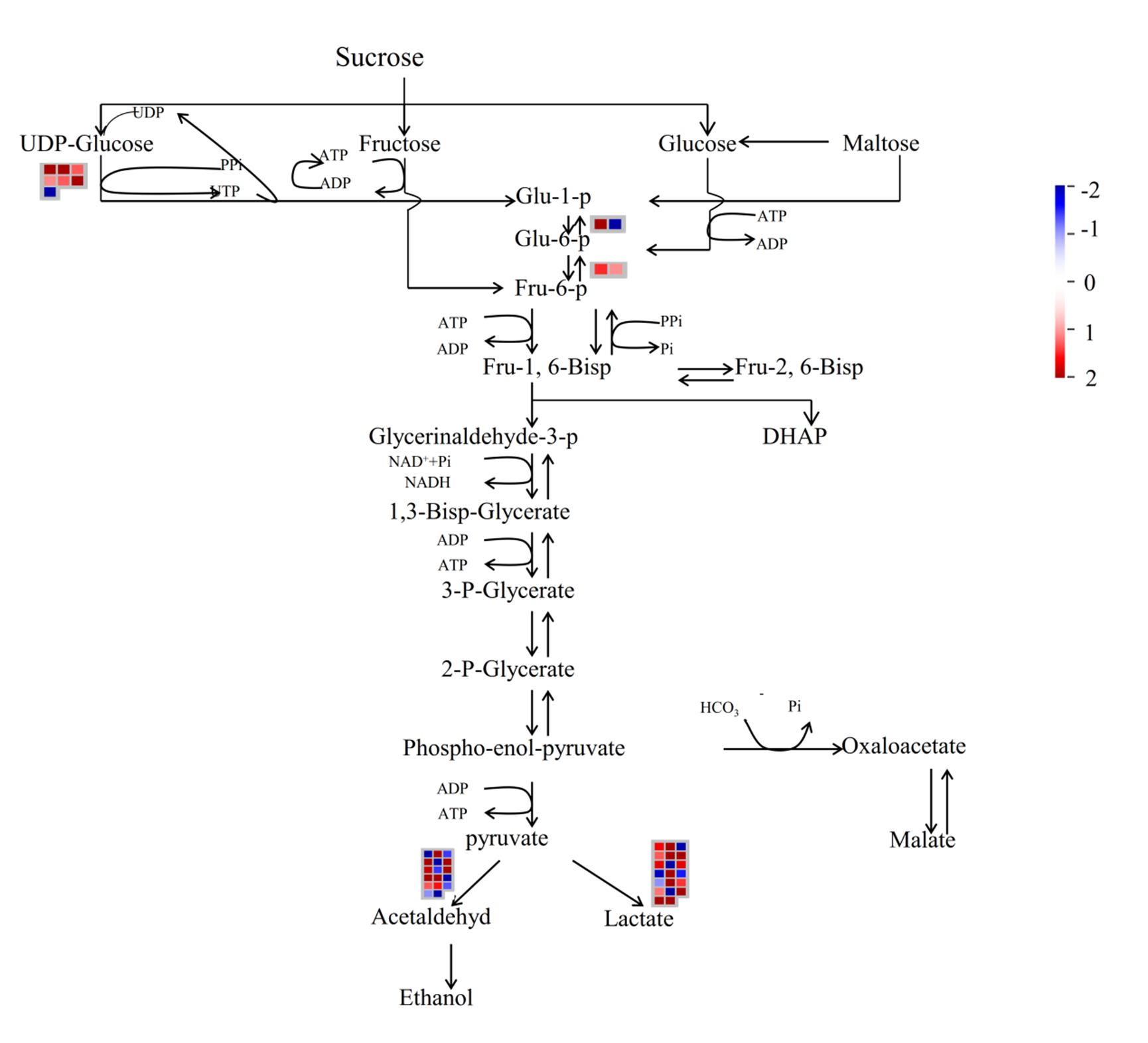

Supplement: Supplementary Figure 8 — DEGs enriched in the plant glycolytic pathway in SL15_25D. [file Image_8.TIF]

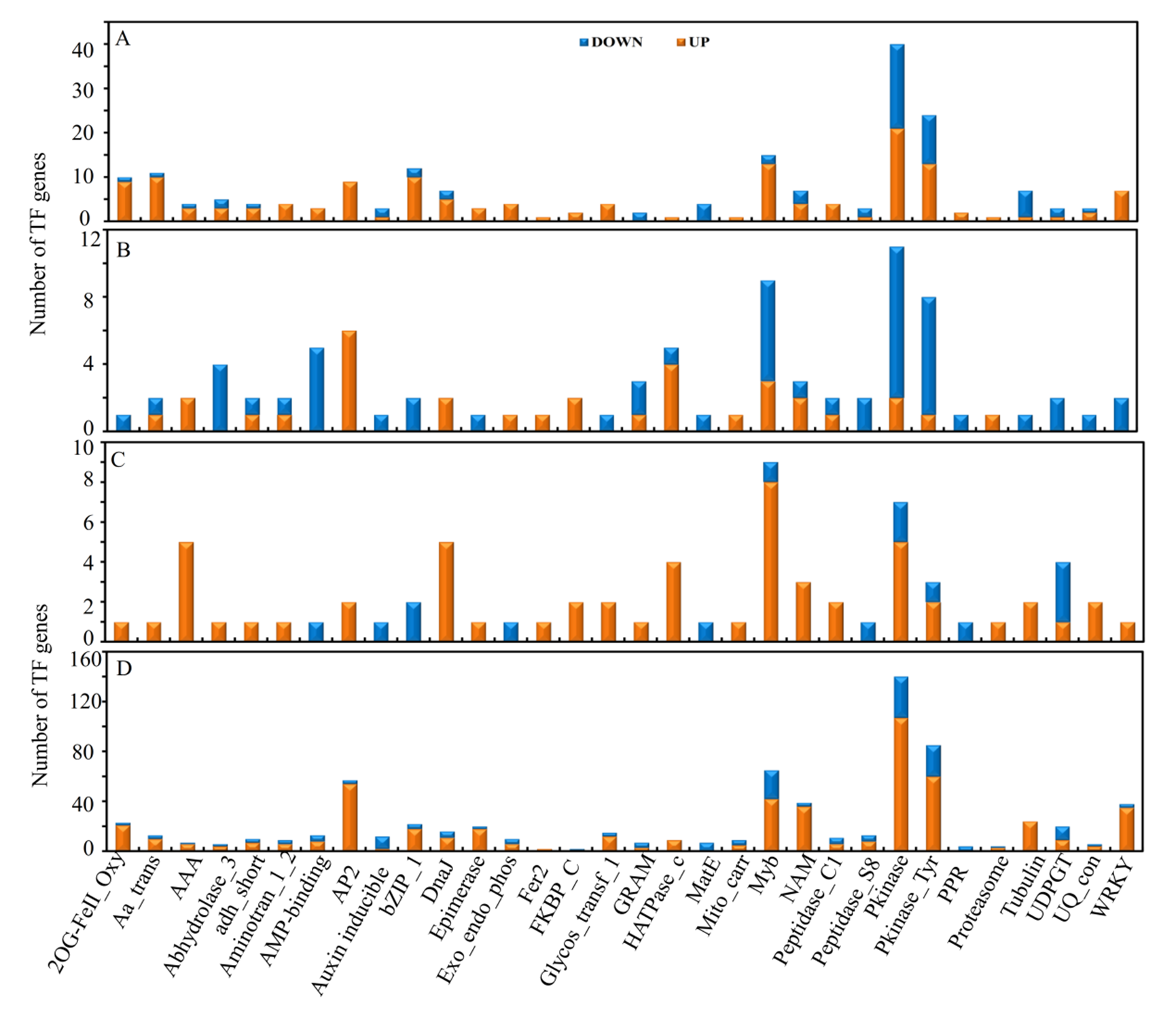

Supplement: Supplementary Figure 9 — Number of DEGs in common TF families that showed up- or downregulation between SL15 and LMY22 during fiber development: (A) 10 DPA, (B) 15 DPA, (C) 20 DPA, and (D) 25 DPA. [file Image_9.TIF]

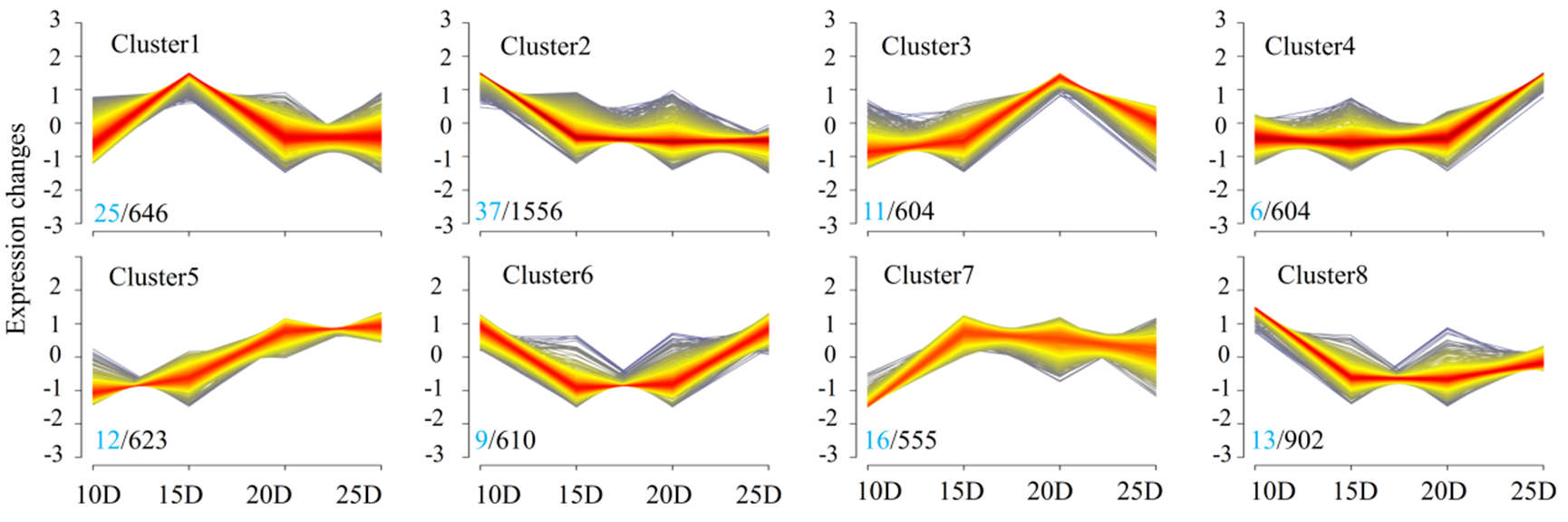

Supplement: Supplementary Figure 10 — Expression pattern of candidate genes in SL15 during different fiber development periods. Blue and black numbers respectively count genes in the substituted chromosome segment and DEGs in each cluster. [file Image_10.TIF]

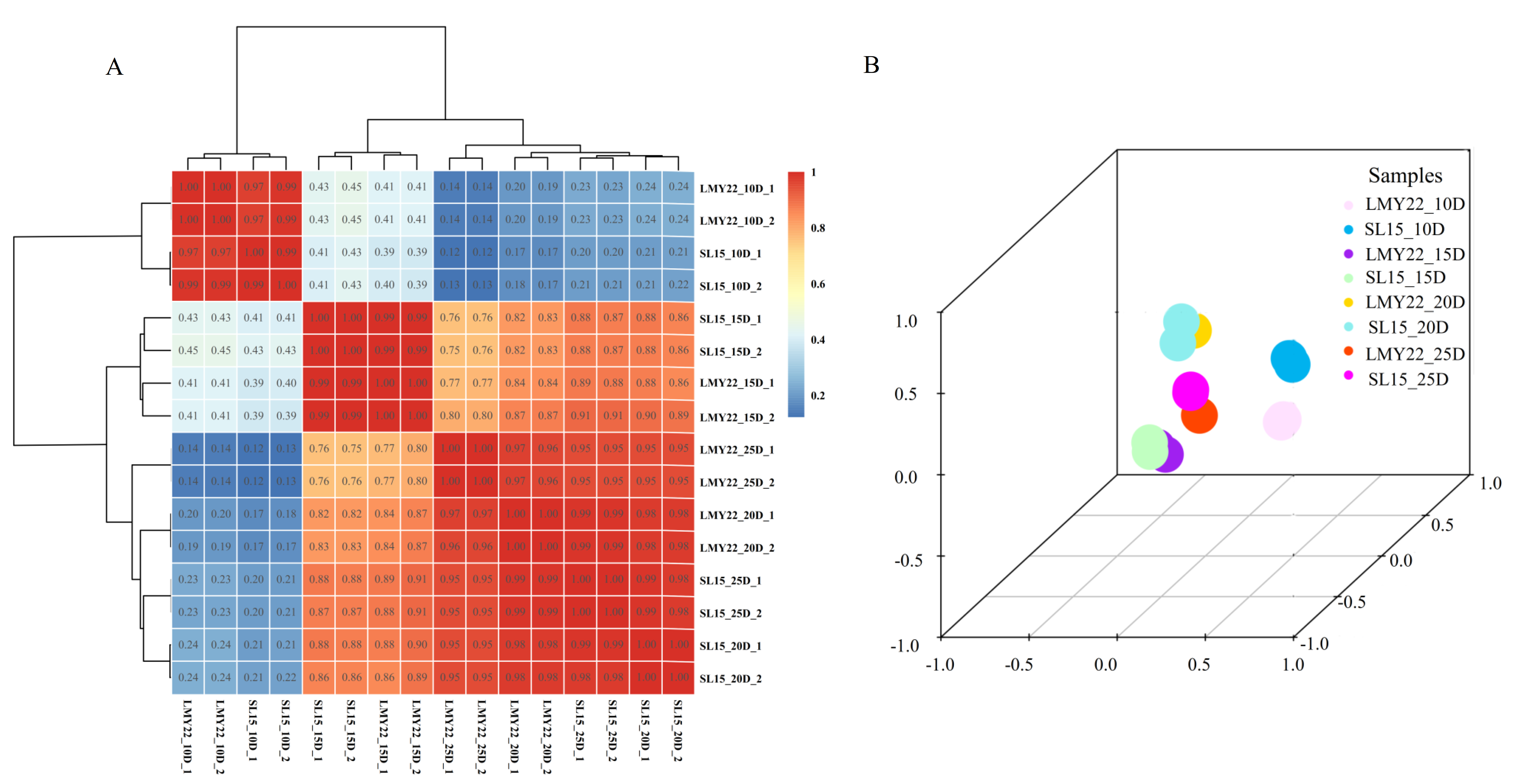

Supplement: Supplementary Figure 11 — Transcriptome correlation and clustering analyses. (A) Spearman correlation coefficient (SCC) analysis of LMY22 and SL15 transcriptomes across fiber development periods. (B) Principal component analysis (PCA) plot showing clustering in LMY22 and SL15 transcriptomes during four fiber development periods. [file Image_11.TIF]
